# Planned mode of birth after previous caesarean section and women's use of psychotropic medication in the first year postpartum: a population-based record linkage cohort study

**DOI:** 10.1017/S0033291720005322

**Published:** 2022-10

**Authors:** Kathryn E. Fitzpatrick, Maria A. Quigley, Daniel J. Smith, Jennifer J. Kurinczuk

**Affiliations:** 1 National Perinatal Epidemiology Unit, Nuffield Department of Population Health, University of Oxford, Oxford, UK; 2 Institute of Health and Wellbeing, University of Glasgow, Glasgow, UK

**Keywords:** Elective repeat caesarean section (ERCS), Vaginal birth after previous caesarean (VBAC), Trial of labor after previous caesarean (TOLAC), Maternal mental health, Postnatal mental health, Psychotropic medication

## Abstract

**Background:**

Policy in many high-income settings supports giving pregnant women with previous caesarean section a choice between an elective repeat caesarean section (ERCS) or planning a vaginal birth after previous caesarean (VBAC), provided they have no contraindications to VBAC. Despite the potential for this choice to influence women's mental health, evidence about the associated effect to counsel women and identify potential targets for intervention is limited. This study investigated the association between planned mode of birth after previous caesarean and women's subsequent use of psychotropic medications.

**Methods:**

A population-based cohort study of 31 131 women with one or more previous caesarean sections who gave birth to a term singleton in Scotland between 2010 and 2015 with no prior psychotropic medications in the year before birth was conducted using linked Scottish national datasets. Cox regression was used to investigate the association between planned mode of birth and being dispensed psychotropic medications in the first year postpartum adjusted for socio-demographic, medical, pregnancy-related factors and breastfeeding.

**Results:**

Planned VBAC (*n* = 10 220) compared to ERCS (*n* = 20 911) was associated with a reduced risk of the mother being dispensed any psychotropic medication [adjusted hazard ratio (aHR) 0.85, 95% confidence interval (CI) 0.78–0.92], an antidepressant (aHR 0.83, 95% CI 0.76–0.90), and at least two consecutive antidepressants (aHR 0.83, 95% CI 0.75–0.91) in the first year postpartum.

**Conclusions:**

Women giving birth by ERCS were more likely than those having a planned VBAC to be dispensed psychotropic medication including antidepressants in the first year postpartum. Further research is needed to establish the reasons behind this new finding.

## Introduction

Caesarean section rates have increased in many parts of the world (Betran et al., 2016), including the UK where around 30% of all births now occur by caesarean (Information Services Division Scotland, 2019; NHS Digital, 2019; Welsh Government, 2020). This has resulted in increasing numbers of pregnant women with a history of previous caesarean. Policy in many high-income countries supports giving such women a choice between planning another caesarean, known as an elective repeat caesarean section (ERCS) or planning a vaginal birth, known as a planned vaginal birth after previous caesarean (VBAC), provided they have no contraindications to VBAC. This contrasts with the general obstetric population in most high-income settings, where the majority of caesareans are performed because they are recommended for various obstetric or medical reasons. Clinical guidelines (American College of Obstetricians & Gynecologists, 2019; National Institute for Health & Clinical Excellence, 2011; Royal College of Obstetricians and Gynaecologists, 2015; The Royal Australian & New Zealand College of Obstetricians & Gynaecologists, 2015) advocate counselling women about the risks and benefits of planned VBAC compared to ERCS to help them make an informed decision about this choice. However, several significant limitations have been highlighted with the existing evidence (Guise et al., 2010). These include uncertainty around whether women who would not have been advised to plan a VBAC due to obstetric/medical reasons were included in the ERCS group. There also remains a lack of evidence about certain outcomes, including the effect of planned mode of birth after previous caesarean on women's mental health.

Concerns have been raised that caesarean section may adversely affect women's mental health by, for example, restricting perceived control over birth, violating expectations about childbirth, disrupting physiological hormonal processes and leading to a more painful and difficult postnatal recovery (Buckley, 2015; Lobel & DeLuca, 2007). However, studies examining the association between mode of birth and women's mental health have reported mixed findings (Ayers, Bond, Bertullies, & Wijma, 2016; Carter, Frampton, & Mulder, 2006; Kendell, Chalmers, & Platz, 1987; Valdimarsdottir, Hultman, Harlow, Cnattingius, & Sparen, 2009). This may partly reflect limitations with the existing studies, with many comprising small non-population-based studies that have focused on the effect of actual mode of birth without differentiating between planned and emergency caesarean despite the very different contexts in which these two types of caesarean are performed. Most previous studies have also not considered potential effects on mental health problems besides postnatal depression. Also, to our knowledge, only one small study of under 300 women (Law et al., 2010) conducted in a single hospital has to date specifically investigated the effect of planned mode of birth after previous caesarean on women's mental health, reporting no significant effects. Studies conducted in the general obstetric population may not be applicable to the situation of birth after previous caesarean as they are more prone to confounding by indication, where adverse outcomes may be a consequence of the underlying causes that led to the need for the caesarean. Furthermore, as certain serious birth-related complications such as uterine rupture, which may adversely affect women's mental health, predominately only occur or are increased following a planned VBAC (Fitzpatrick et al., 2012; Fitzpatrick, Kurinczuk, Bhattacharya, & Quigley, 2019), planned VBAC compared to ERCS may actually be associated with poorer subsequent maternal mental health.

Apart from providing further information to counsel women, the potential profound impact of maternal mental health problems on the woman, her infant and other family members (Slomian, Honvo, Emonts, Reginster, & Bruyere, 2019), makes it important to better understand the aetiology and triggers for such problems to help identify potential targets for intervention. The aim of this study was to investigate the association between planned mode of birth after previous caesarean section and women's use of psychotropic medication in the first year postpartum as an indication of their subsequent risk of having treated mental health problems, among women giving birth to a singleton at term and considered clinically eligible to plan a VBAC.

## Methods

### Study design and data sources

A population-based cohort study was conducted using Scottish national datasets (online Supplementary Table S1) linked using exact matching of the community health index number, a unique person identifier used in Scotland. Online Supplementary Table S2 details the data sources, codes and database fields used.

### Study population

All women with one or more previous caesareans who gave birth to a term (37–41 completed weeks gestation) singleton in Scotland, UK, between 1st January 2010 and 31st December 2015 were identified. Births to women not considered clinically eligible to plan a VBAC based on UK guidelines (National Institute for Health and Clinical Excellence, 2011; Royal College of Obstetricians and Gynaecologists, 2015) were excluded (online Supplementary Table S2). Births to women who had an antepartum stillbirth were also excluded as vaginal birth is usually recommended in this situation (Royal College of Obstetricians and Gynaecologists, 2010). To identify incident events of psychotropic drug use, women dispensed any of the primary outcome medications in the year before they gave birth were excluded. Women migrating to Scotland less than 1 year before they gave birth were also excluded to ensure information on history of psychotropic medication for at least 1 year before birth. Additional exclusions included stillbirths missing information about whether they occurred antenatally or during labour or birth, births missing information on mode of birth or gestational age at birth, births by non-elective caesarean section missing information about duration of labour, and births to women whose number of previous caesarean sections was greater than their recorded parity (online Supplementary Fig. S1). Each woman was followed up from the date they gave birth until the outcome of interest, date of emigration, death or 1 year after they gave birth, whichever came first.

### Exposures

The primary exposure of interest was planned mode of birth after previous caesarean, with planned VBAC (birth vaginally or by non-elective caesarean with a duration of labour of ⩾1 h) compared to ERCS (birth by elective caesarean, defined by the Information Services Division Scotland as a caesarean performed during the day with both the patient and staff fully prepared).

Analysis was also performed according to whether planned VBAC was attempted with or without labour induction compared to ERCS, and was conducted according to actual mode of birth after previous caesarean, defined as follows: women recorded as having a vaginal birth were classified as having a VBAC; women recorded as having a non-elective caesarean with a duration of labour of ⩾1 h were classified as having an in-labour non-elective repeat caesarean and women recorded as having an elective caesarean were classified as having an ERCS.

### Outcomes

The primary outcome was time to first dispensed prescription for any psychotropic medication within the first year postpartum, including medicines in any of the following ‘legacy’ British National Formulary (BNF) sections (Information Services Division Scotland, 2018): 4.3 ‘Antidepressant drugs’, used to treat a range of affective symptomatology including depressive disorders, anxiety disorders and mixed symptomatology; 4.1.1 ‘Hypnotics’, used to treat insomnia; 4.1.2 ‘Anxiolytics’, used to treat anxiety and 4.2 ‘Drugs used in psychoses and related disorders’.

Secondary outcomes included time to first dispensed prescription within the first year postpartum for specific categories of psychotropic medications, analysing antidepressants (BNF 4.3), hypnotics and/or anxiolytics (BNF 4.1.1 and/or 4.1.2) and drugs used in psychoses and related disorders (BNF 4.2) as three separate outcomes. We also examined at least two consecutive prescriptions for an antidepressant, where the first antidepressant was dispensed within the first year postpartum followed by at least one additional prescription for an antidepressant within 91 days of the first. Most antidepressants were dispensed over monthly intervals. A 91 day cut-off was used to allow for women decreasing their dosage for a period, using stored medication or late collection of a repeat prescription.

In the absence of data about the reason for prescribing, we attempted to exclude drugs likely to have been prescribed for indications other than mental health problems by excluding tricyclic antidepressants prescribed at a lower dose than the recommended BNF level for the treatment of depression (online Supplementary Table S2), as lower doses may be prescribed for other reasons such as chronic pain.

### Statistical analysis

To investigate the association between the exposures and each outcome, Cox proportional hazards models were used to estimate hazard ratios (HRs) and 95% confidence intervals (CIs). All models were adjusted for year of delivery to account for any temporal changes. Models were then adjusted in a hierarchical fashion for potential confounders determined *a priori*: model A adjusted for socio-demographic factors; model B additionally adjusted for maternal medical and pregnancy-related factors. Confounders were determined *a priori* based on pre-existing hypotheses or evidence on what factors are thought to potentially confound the relationship between the exposure and outcome in question (Field, 2018; Ghaedrahmati, Kazemi, Kheirabadi, Ebrahimi, & Bahrami, 2017; Gregory et al., 2008; Guise et al., 2010; O'Hara & McCabe, 2013), with a conceptual framework of how the factors might influence the relationship between planned mode of birth after previous caesarean and the outcomes considered outlined in online Supplementary Fig. S2. Breastfeeding may be on the causal pathway in that the exposures are associated with a woman's likelihood of breastfeeding (Fitzpatrick et al., 2019), and breastfeeding may in turn influence a woman's likelihood of being dispensed psychotropic medication (Borra, Iacovou, & Sevilla, 2015; Tripathi & Majumder, 2010). As we were interested in the effects of the exposures that were not mediated through breastfeeding an additional analysis was performed adjusting for whether the mother breastfed (exclusively or mixed) around 6–8 weeks postpartum (model C). We also examined if there was evidence of effect modification between the exposures and any breastfeeding at 6–8 weeks postpartum, in addition to investigating the number of prior caesareans and any prior vaginal birth as potential effect modifiers of the relationship between the primary exposure and outcomes. Effect modification was investigated by the addition of interaction terms to the full regression models. Adjustments were only performed when there was a minimum of 5–9 outcome events per coefficient in the model (Vittinghoff & McCulloch, 2007).

Fractional polynomials were used to examine whether continuous covariates showed evidence of departure from linearity. A total of 24.7% of the study population has missing data on one or more of the covariates included in the fully adjusted models (see footnotes of Table 1 for more details). Multiple imputation using a recently developed extension to the chained equations approach was used (Bartlett, Seaman, White, & Carpenter, 2014) to impute partially observed covariates when there appeared from the complete case analysis to be evidence of non-linear covariate effects. Otherwise, the normal chained equations method was used to impute partially observed covariates including the event indicator and Nelson–Aalen estimator of the cumulative hazard in the imputation models (White, Royston, & Wood, 2009) in addition to all covariates, and performing 30 imputations. None of the exposures of interest, but one or more of the covariates in some models appeared to violate the proportional hazards assumption assessed using Schoenfeld residuals and log–log plots in the complete case analysis. We decided not to model these covariates as time-varying covariates as this was not found to result in material differences to the exposure estimates. Robust standard errors were used to account for the lack of independence in the data of women who had more than one eligible birth in the study period.

**Table 1.** Characteristics of study cohort by planned mode of birth after previous caesarean section

	ERCS *n* (%)^a^, unless otherwise stated (*n* = 20 911)	Planned VBAC *n* (%)^a^, unless otherwise stated (*n* = 10 220)
**Sociodemographic characteristics**		
Maternal age (years)		
Less than 25	1871 (8.9)	1146 (11.2)
25–29	4756 (22.7)	2510 (24.6)
30–34	7269 (34.8)	3645 (35.7)
35–39	5553 (26.6)	2429 (23.8)
40 or more	1462 (7.0)	490 (4.8)
Median (IQR) maternal age (years)	32 (28–36)	32 (28–35)
Mother's country of birth		
UK	17 900 (85.6)	8406 (82.3)
Non-UK	3011 (14.4)	1814 (17.7)
Marital status/registration type		
Married or joint registration/same address	18 766 (89.7)	9201 (90.0)
Joint registration/different address	1594 (7.6)	726 (7.1)
Sole registration	551 (2.6)	293 (2.9)
Socioeconomic status^b^		
Managerial/professional	10 479 (50.1)	5140 (50.3)
Intermediate	4429 (21.2)	1987 (19.4)
Routine/manual	5261 (25.2)	2611 (25.5)
Other^c^	742 (3.5)	482 (4.7)
**Maternal medical and pregnancy-related characteristics**		
Number of previous caesarean sections		
1	15 996 (76.5)	9821 (96.1)
2 or more	4915 (23.5)	399 (3.9)
Median (IQR) number of previous caesarean sections	1 (1–1)	1 (1–1)
Any prior vaginal birth^d^		
No	17 330 (83.1)	6153 (60.4)
Yes	3517 (16.9)	4039 (39.6)
Parity^d^		
1	13 458 (64.6)	5957 (58.5)
2 or more	7385 (35.4)	4219 (41.5)
Median (IQR) parity^d^	1 (1–2)	1 (1–2)
Interpregnancy interval (months)^d^		
24 or more	11 634 (60.0)	4629 (55.3)
12–23	5192 (26.8)	2441 (29.1)
Less than 12	2568 (13.2)	1307 (15.6)
Median (IQR) interpregnancy interval (months)^d^	29.1 (17.3–48.5)	26.3 (15.8–45.3)
Any prior stillbirth or neonatal death^d^		
No	20 356 (97.6)	10 025 (98.4)
Yes	506 (2.4)	168 (1.7)
Mother smoked at booking^d^		
No	17 295 (86.8)	8241 (83.3)
Yes	2637 (13.2)	1649 (16.7)
Maternal BMI at booking (kg/m^2^)^d^		
Less than 25	7113 (36.7)	4676 (48.5)
25–29.9	5919 (30.5)	2843 (29.5)
30 or more	6374 (32.8)	2128 (22.1)
Median (IQR) BMI at booking (kg/m^2^)^d^	26.9 (23.5–31.9)	25.2 (22.4–29.3)
Any hypertensive disorder	694 (3.3)	430 (4.2)
Pre-existing or gestational diabetes	1081 (5.2)	253 (2.5)
**Intrapartum and postpartum characteristics**		
Adverse perinatal outcome^d^^,^^e^		
No	18 065 (94.7)	8956 (93.9)
Yes	1020 (5.3)	580 (6.1)
Maternal intrapartum or postpartum complication^f^		
No	19 901 (95.2)	9270 (90.7)
Yes	1010 (4.8)	950 (9.3)
Any breastfeeding at 6–8 week review^d^		
No	12 208 (63.6)	5007 (52.5)
Yes	6990 (36.4)	4525 (47.5)

BMI, body mass index; ERCS, elective repeat caesarean section; IQR, interquartile range; NS-SEC, National Statistics Socio-Economic Classification; VBAC, vaginal birth after previous caesarean.

a Percentage of those with complete data.

b Socioeconomic status of mother for sole registered birth or highest of mother's or father's socioeconomic status for births registered inside marriage or jointly registered by both parents outside marriage. Socioeconomic status defined by NS-SEC based on occupation and employment status.

c Other includes never worked/long-term unemployed, student, not stated or not classifiable.

d Missing data: any prior vaginal birth 92 (0.30%); parity 112 (0.36%); interpregnancy interval 3360 (10.8%); any prior stillbirth or neonatal death 76 (0.24%); maternal smoking status 1309 (4.20%); maternal BMI 2078 (6.68%); adverse perinatal outcome 2510 (8.77%); any breastfeeding at 6–8 week review 2401 (7.71%).

e Adverse perinatal outcome includes intrapartum stillbirth or neonatal death, admission to a neonatal unit, resuscitation with drugs and/or intubation or an Apgar score <7 at 5 min.

f Intrapartum or postpartum complication includes uterine rupture, peripartum hysterectomy, blood transfusion, puerperal sepsis, other puerperal infection, surgical injury (damage to bowel, bladder or ureter requiring surgical repair), third- or fourth-degree perineal tear or overnight readmission to hospital within 42 days of birth.

A number of sensitivity analyses were conducted. First, a complete case analysis for each outcome studied. Second, given the potential of the way we defined planned mode of birth to misclassify women who planned ERCS but went into labour before their scheduled caesarean date, analyses were repeated restricted to births at ⩾39 weeks gestation. Since 2004, this is the gestation recommended by UK guidelines to perform an ERCS (National Institute for Health & Clinical Excellence, 2011; Royal College of Obstetricians & Gynaecologists, 2015). Third, analyses were repeated confined to women without a history of being dispensed any of the primary outcome medications in the 2 years before they gave birth. As the prescription information was only available from 2009, only eligible women who gave birth between 1st January 2011 and 31st December 2015 and did not migrate to Scotland less than 2 years before they gave birth were included in this analysis. Fourth, *E*-values were calculated to quantify the minimum strength of association that an unmeasured confounder would need with both the primary exposure and a given outcome to fully explain any observed associations between the primary exposure and outcomes conditional on the measured covariates (VanderWeele & Ding, 2017). All analyses were conducted in StataMP version 14 or 16.

### Ethical approval

The study did not require ethics committee approval as it involved secondary analysis of anonymised data. However, approval for the study was obtained from the Public Benefit and Privacy Panel for Health and Social Care Scotland (application number 1516-0196).

## Results

A total of 31 131 singleton term births to women with one or more previous caesareans met the study eligibility criteria (online Supplementary Fig. S1). Overall, 32.8% (10 220) of the women had a planned VBAC and 67.2% (20 911) had an ERCS. The ERCS rate increased over the study period from 64.5% in 2010 to 71.9% in 2015. Women who had a planned VBAC were more likely than those who had an ERCS to be younger, born outside the UK and have a lower socio-economic status (Table 1). They were also more likely to have had just one prior caesarean section, one or more previous vaginal births, a shorter inter-pregnancy interval, be smokers at booking for pregnancy care, have a hypertensive disorder, experienced an adverse perinatal outcome or maternal intrapartum or postpartum complication and have breastfed at 6–8 weeks postpartum. They were less likely than those who had an ERCS to have experienced a prior stillbirth or neonatal death, be overweight or obese or to have diabetes.

Less than 1% of the study population was lost to follow-up because of emigration or death, with these women contributing person-years up to the date of loss. During 29 097 person-years of follow-up, 3585 (11.5%) of the study population were dispensed at least one prescription for any psychotropic medication. Antidepressants were the most common type of psychotropic medication dispensed, with 9.9% of women dispensed an antidepressant, 2.9% dispensed a hypnotic and/or anxiolytic and 0.20% dispensed an antipsychotic and/or related drug. Crude rates of first dispensed prescription for any psychotropic medication, an antidepressant and at least two consecutive antidepressants peaked around month 2 to 3 following childbirth, whereas crude rates of first dispensed prescription for a hypnotic and/or anxiolytic or an antipsychotic and/or related drug remained fairly constant over the follow-up period (online Supplementary Fig. S3).

**Fig. 1.**
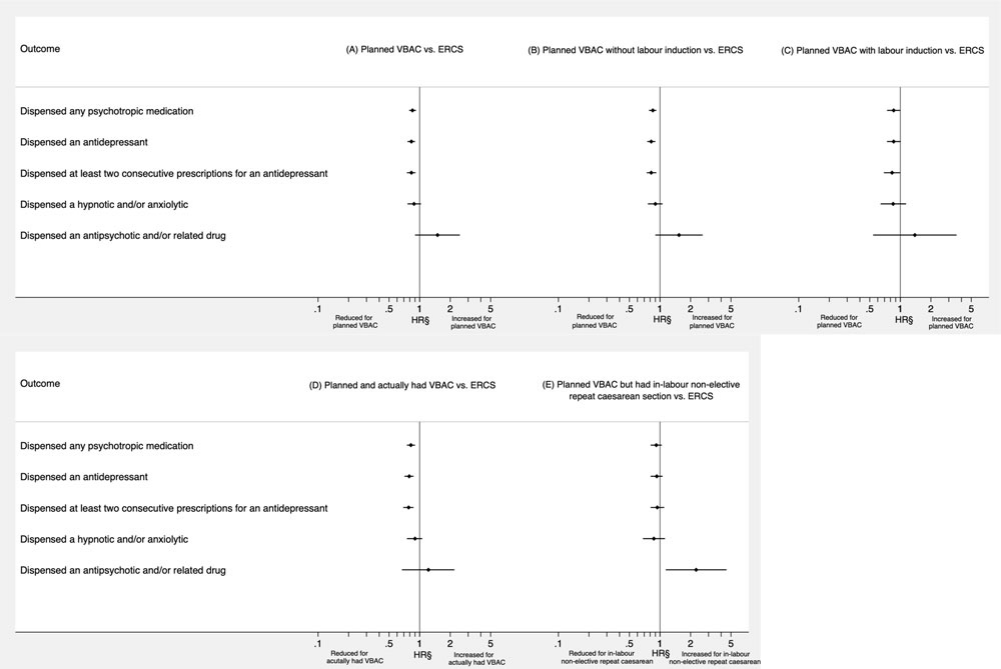
Outcomes following (*a*) planned VBAC compared to ERCS, (*b*) planned VBAC without labour induction compared to ERCS, (*c*) planned VBAC with labour induction compared to ERCS, (*d*) planned and actually had VBAC compared to ERCS and (*e*) planned VBAC but had in-labour non-elective repeat caesarean section compared to ERCS. ^§^Dispensed an antipsychotic and/or related drug only adjusted for year of delivery and socio-demographic factors (maternal age, mother's country of birth, marital status and socio-economic status) because of low number of events, whereas other outcomes were adjusted for year of delivery and socio-demographic factors (maternal age, mother's country of birth, marital status and socio-economic status), maternal medical and pregnancy-related factors (number of previous caesarean sections, any prior vaginal birth, inter-pregnancy interval, any prior stillbirth or neonatal death, maternal smoking status at booking, maternal BMI at booking, hypertensive disorder and diabetes) and breastfeeding at 6–8 weeks postpartum. BMI, body mass index; ERCS, elective repeat caesarean section; HR, hazard ratio; VBAC, vaginal birth after previous caesarean.

Outcomes according to planned mode of birth are shown in Table 2 and Fig. 1*a*. Having only adjusted for year of delivery, women who planned a VBAC had a significantly lower risk than those giving birth by ERCS of being dispensed any psychotropic medication (HR 0.80, 95% CI 0.75–0.87) and all categories of psychotropic medication (antidepressant: HR 0.78, 95% CI 0.72–0.85; at least two consecutive antidepressants: HR 0.76, 95% CI 0.69–0.83 and hypnotic and/or anxiolytic 0.85, 95% 0.74–0.99) except for antipsychotic and/or related drugs (HR 1.54, 95% CI 0.94–2.52). Adjustment for socio-economic factors and maternal medical and pregnancy-related factors had little material effect on the effect estimates. Adjusting for any breastfeeding at 6–8 weeks postpartum slightly attenuated all the effect estimates, but the risk of being dispensed any psychotropic medication [adjusted HR (aHR) 0.85, 95% CI 0.78–0.92], an antidepressant (aHR 0.83, 95% CI 0.76–0.90) and at least two consecutive antidepressants (aHR 0.83, 95% CI 0.75–0.91) remained significantly lower in women who planned a VBAC compared to giving birth by ERCS.

**Table 2.** Outcomes following planned VBAC compared to ERCS

Outcomes	ERCS	Planned VBAC	Base model^a^ HR (95% CI)	Model A^b^ HR (95% CI)	Model B^c^ HR (95% CI)	Model C^d^ HR (95% CI)
	Number of events/person-years (rate per 100 person-years)	Number of events/person-years (rate per 100 person-years)				
Dispensed any psychotropic medication	2565/19 459 (13.18)	1020/9638 (10.58)	**0.80 (0.75–0.87)**	**0.80 (0.75–0.86)**	**0.80 (0.74–0.87)**	**0.85 (0.78–0.92)**
			***p* < 0.001**	***p* < 0.001**	***p* < 0.001**	***p* < 0.001**
Dispensed an antidepressant	2228/19 620 (11.36)	859/9722 (8.84)	**0.78 (0.72–0.85)**	**0.78 (0.72–0.84)**	**0.78 (0.72–0.85)**	**0.83 (0.76–0.90)**
			***p* < 0.001**	***p* < 0.001**	***p* < 0.001**	***p* < 0.001**
Dispensed at least two consecutive prescriptions for an antidepressant	1702/19 913 (8.55)	633/9843 (6.43)	**0.76 (0.69–0.83)**	**0.76 (0.69–0.83)**	**0.77 (0.70–0.85)**	**0.83 (0.75–0.91)**
			***p* < 0.001**	***p* < 0.001**	***p* < 0.001**	***p* < 0.001**
Dispensed a hypnotic and/or anxiolytic	637/20 535 (3.10)	267/10 040 (2.66)	**0.85 (0.74–0.99)**	0.87 (0.75–1.00)	**0.83 (0.71–0.97)**	0.88 (0.76–1.03)
			***p* = 0.032**	*p* = 0.054	***p* = 0.021**	*p* = 0.119
Dispensed an antipsychotic and/or related drug	36/20 800 (0.17)	27/10 152 (0.27)	1.54 (0.94–2.52)	1.50 (0.90–2.49)	NC	NC
			*p* = 0.088	*p* = 0.116		

BMI, body mass index;CI, confidence interval; ERCS, elective repeat caesarean section; HR, hazard ratio; NC, not calculated because of low number of events; VBAC, vaginal birth after previous caesarean.

a Base model adjusted for year of delivery only.

b Model A adjusted for year of delivery and socio-demographic factors (maternal age, mother's country of birth, marital status and socio-economic status).

c Model B adjusted for variables in model A and additionally adjusted for maternal medical and pregnancy-related factors (number of previous caesarean sections, any prior vaginal birth, inter-pregnancy interval, any prior stillbirth or neonatal death, maternal smoking status at booking, maternal BMI at booking, hypertensive disorder and diabetes).

d Model C, adjusted for variables in model B and additionally adjusted for any breastfeeding at 6–8 weeks postpartum.

Bold text indicates statistically significant findings at the 5% level.

Of the women who planned a VBAC, 19.3% (1960/10 180) had their labour induced (30.3% using surgical induction alone, 33.5% using medical induction alone and 34.7% using surgical and medication induction). Outcomes by whether planned VBAC was attempted with or without labour induction compared to ERCS (Table 3 and Fig. 1*b* and *c*) were very similar to those reported for planned VBAC compared to ERCS. Compared to the women who gave birth by ERCS, women who planned a VBAC with or without labour induction had a significantly reduced risk of being dispensed any psychotropic medication even having adjusted for breastfeeding (aHR 0.86, 95% CI 0.74–1.00 and aHR 0.85, 95% CI 0.78–0.92, respectively). There was also evidence that women who planned a VBAC with or without labour induction had a reduced risk of being dispensed an antidepressant and at least two consecutive antidepressants. However, for women who planned a VBAC with labour induction these effects were not statistically significant having adjusted for breastfeeding noting the smaller number of women in this subgroup.

**Table 3.** Outcomes following planned VBAC with and without labour induction compared to ERCS

	ERCS	Planned VBAC without labour induction					Planned VBAC with labour induction				
Outcomes	Number of events/person-years (rate per 100 person-years)	Number of events/person-years (rate per 100 person-years)	Base model^a^ HR (95% CI)	Model A^b^ HR (95% CI)	Model B^c^ HR (95% CI)	Model C^d^ HR (95% CI)	Number of events/person-years (rate per 100 person-years)	Base model^a^ HR (95% CI)	Model A^b^ HR (95% CI)	Model B^c^ HR (95% CI)	Model C^d^ HR (95% CI)
Dispensed any psychotropic medication	2565/19 459	805/7758	**0.79**	**0.80**	**0.80**	**0.85**	212/1841	0.87	**0.83**	**0.81**	**0.86**
	(13.18)	(10.38)	**(0.73–0.85)**	**(0.73–0.86)**	**(0.74–0.87)**	**(0.78–0.92)**	(11.51)	(0.76–1.01)	**(0.72–0.96)**	**(0.70–0.94)**	**(0.74–1.00)**
			***p* < 0.001**	***p* < 0.001**	***p* < 0.001**	***p* < 0.001**		*p* = 0.060	***p* = 0.012**	***p* = 0.005**	***p* = 0.046**
Dispensed an antidepressant	2228/19 620	673/7825	**0.76**	**0.76**	**0.77**	**0.82**	183/1858	0.87	**0.82**	**0.81**	0.86
	(11.36)	(8.60)	**(0.70–0.83)**	**(0.70–0.83)**	**(0.70–0.85)**	**(0.75–0.90)**	(9.85)	(0.74–1.01)	**(0.71–0.96)**	**(0.69–0.95)**	(0.74–1.01)
			***p* < 0.001**	***p* < 0.001**	***p* < 0.001**	***p* < 0.001**		*p* = 0.065	***p* = 0.011**	***p* = 0.009**	*p* = 0.065
Dispensed at least two consecutive prescriptions for an antidepressant	1702/19 913	499/7915	**0.74**	**0.75**	**0.77**	**0.82**	132/1889	**0.82**	**0.78**	**0.78**	0.83
	(8.55)	(6.30)	**(0.67–0.82)**	**(0.68–0.83)**	**(0.69–0.86)**	**(0.74–0.92)**	(6.99)	**(0.68–0.98)**	**(0.66–0.94)**	**(0.65–0.94)**	(0.69–1.00)
			***p* < 0.001**	***p* < 0.001**	***p* < 0.001**	***p* < 0.001**		***p* = 0.026**	***p* = 0.008**	***p* = 0.008**	*p* = 0.055
Dispensed a hypnotic and/or anxiolytic	637/20 535	214/8074	**0.85**	0.87	**0.85**	0.90	53/1926	0.89	0.87	0.80	0.85
	(3.10)	(2.65)	**(0.73–0.99)**	(0.75–1.02)	**(0.72–1.00)**	(0.76–1.06)	(2.75)	(0.67–1.17)	(0.66–1.15)	(0.60–1.07)	(0.64–1.14)
			***p* = 0.042**	*p* = 0.088	***p* = 0.048**	*p* = 0.200		*p* = 0.405	*p* = 0.332	*p* = 0.137	*p* = 0.289
Dispensed an antipsychotic and/or related drug	36/20 800	22/8161	1.56	1.54	NC	NC	5/1951	1.48	1.39	NC	NC
	(0.17)	(0.27)	(0.92–2.64)	(0.90–2.63)			(0.26)	(0.58–3.78)	(0.54–3.58)		
			*p* = 0.098	*p* = 0.117				*p* = 0.412	*p* = 0.493		

BMI, body mass index; CI, confidence interval; ERCS, elective repeat caesarean section; HR, hazard ratio; NC, not calculated because of low number of events; VBAC, vaginal birth after previous caesarean.

a Base model adjusted for year of delivery only.

b Model A adjusted for year of delivery and socio-demographic factors (maternal age, mother's country of birth, marital status and socio-economic status).

c Model B adjusted for variables in model A and additionally adjusted for maternal medical and pregnancy-related factors (number of previous caesarean sections, any prior vaginal birth, inter-pregnancy interval, any prior stillbirth or neonatal death, maternal smoking status at booking, maternal BMI at booking, hypertensive disorder and diabetes).

d Model C, adjusted for variables in model B and additionally adjusted for any breastfeeding at 6–8 weeks postpartum.

Bold text indicates statistically significant findings at the 5% level.

Of the women who planned a VBAC, 71.6% (7314/10 220) went on to have a VBAC and 28.4% (2906/10 220) went on to have an in-labour non-elective repeat caesarean. Compared to the women who gave birth by ERCS, both the women who actually had a VBAC and the women who gave birth by in-labour non-elective repeat caesarean had a reduced risk of being dispensed any psychotropic medication, an antidepressant and at least two consecutive antidepressants. However, for women who had an in-labour non-elective repeat caesarean the effect sizes were weaker and not statistically significant in the fully adjusted models (Table 4 and Fig. 1*d* and *e*). Women giving birth by in-labour non-elective repeat caesarean had a significantly increased risk compared to those giving birth by ERCS of being dispensed an antipsychotic and/or related drug having adjusted for year of delivery alone or year of delivery in addition to socio-demographic factors. However, due to the low number of outcome events it was not possible to adjust for additional factors that may influence this association.

**Table 4.** Outcomes according to actual mode of birth – planned and actually had a VBAC and planned VBAC but had in-labour non-elective repeat caesarean section compared to ERCS

	ERCS	Planned and actually had VBAC					Planned VBAC but had in-labour non-elective repeat caesarean section				
Outcomes	Number of events/person-years (rate per 100 person-years)	Number of events/person-years (rate per 100 person-years)	Base model^a^ HR (95% CI)	Model A^b^ HR (95% CI)	Model B^c^ HR (95% CI)	Model C^d^ HR (95% CI)	Number of events/person-years (Rate per 100 person-years)	Base model^a^ HR (95% CI)	Model A^b^ HR (95% CI)	Model B^c^ HR (95% CI)	Model C^d^ HR (95% CI)
Dispensed any psychotropic medication	2565/19 459	725/6900	**0.80**	**0.79**	**0.78**	**0.82**	295/2738	**0.82**	**0.85**	**0.86**	0.92
	(13.18)	(10.51)	**(0.73–0.87)**	**(0.72–0.85)**	**(0.71–0.85)**	**(0.75–0.90)**	(10.77)	**(0.73–0.92)**	**(0.75–0.96)**	**(0.76–0.97)**	(0.81–1.04)
			***p* < 0.001**	***p* < 0.001**	***p* < 0.001**	***p* < 0.001**		***p* = 0.001**	***p* = 0.007**	***p* = 0.013**	*p* = 0.163
Dispensed an antidepressant	2228/19 620	600/6961	**0.76**	**0.75**	**0.74**	**0.79**	259/2760	**0.83**	**0.85**	**0.87**	0.93
	(11.36)	(8.62)	**(0.70–0.84)**	**(0.68–0.82)**	**(0.67–0.82)**	**(0.71–0.87)**	(9.38)	**(0.73–0.94)**	**(0.75–0.97)**	**(0.76–0.99)**	(0.81–1.06)
			***p* < 0.001**	***p* < 0.001**	***p* < 0.001**	***p* < 0.001**		***p* = 0.004**	***p* = 0.017**	***p* = 0.031**	*p* = 0.268
Dispensed at least two consecutive prescriptions for an antidepressant	1702/19 913	436/7053	**0.73**	**0.72**	**0.73**	**0.78**	197/2790	**0.83**	0.86	0.87	0.94
	(8.55)	(6.18)	**(0.66–0.81)**	**(0.65–0.80)**	**(0.65–0.82)**	**(0.69–0.87)**	(7.06)	**(0.71–0.96)**	(0.74–1.00)	(0.75–1.01)	(0.81–1.10)
			***p* < 0.001**	***p* < 0.001**	***p* < 0.001**	***p* < 0.001**		***p* = 0.013**	*p* = 0.052	*p* = 0.077	*p* = 0.437
Dispensed a hypnotic and/or anxiolytic	637/20 535	198/7181	0.89	0.88	0.84	0.90	69/2859	**0.78**	0.83	0.81	0.87
	(3.10)	(2.76)	(0.75–1.04)	(0.75–1.04)	(0.71–1.00)	(0.75–1.06)	(2.41)	**(0.61–1.00)**	(0.64–1.06)	(0.63–1.05)	(0.68–1.12)
			*p* = 0.137	*p* = 0.135	*p* = 0.053	*p* = 0.189		***p* = 0.046**	*p* = 0.130	*p* = 0.109	*p* = 0.283
Dispensed an antipsychotic and/or related drug	36/20 800	16/7264	1.27	1.22	NC	NC	11/2888	**2.20**	**2.27**	NC	NC
	(0.17)	(0.22)	(0.71–2.28)	(0.67–2.20)			(0.38)	**(1.12–4.32)**	**(1.14–4.52)**		
			*p* = 0.417	*p* = 0.512				***p* = 0.022**	***p* = 0.020**		

BMI, body mass index; CI, confidence interval; ERCS, elective repeat caesarean section; HR, hazard ratio; NC, not calculated because of low number of events; VBAC, vaginal birth after previous caesarean.

a Base model adjusted for year of delivery only.

b Model A adjusted for year of delivery and socio-demographic factors (maternal age, mother's country of birth, marital status and socio-economic status).

c Model B adjusted for variables in model A and additionally adjusted for maternal medical and pregnancy-related factors (number of previous caesarean sections, any prior vaginal birth, inter-pregnancy interval, any prior stillbirth or neonatal death, maternal smoking status at booking, maternal BMI at booking, hypertensive disorder and diabetes).

d Model C, adjusted for variables in model B and additionally adjusted for any breastfeeding at 6–8 weeks postpartum.

Bold text indicates statistically significant findings at the 5% level.

Examining the associations by breastfeeding status at 6–8 weeks postpartum, the effects of planned VBAC, planned VBAC without labour induction and actual VBAC were still evident in women who did and did not breastfeed with no evidence of effect modification between the exposures and breastfeeding status (online Supplementary Fig. S4). There was also no evidence of effect modification between the primary exposure and the number or prior caesareans a woman had, or whether the woman had any prior vaginal birth. The sensitivity analyses presented in online Supplementary Tables S3–S11 resulted in similar effect estimates, although the significance of some of the associations varied noting these analyses included a smaller number of women. The *E*-values suggest that relatively modest unmeasured confounding could fully explain the observed associations between the primary exposure and outcomes (online Supplementary Table S12). For example, to fully explain the observed HR of 0.85 for any psychotropic medication, an unmeasured confounder would need to be associated with at least a 1.63-fold increased risk for both the primary exposure and outcome through pathways independent of the covariates included in the fully adjusted model; to move the upper CI to include the null, an unmeasured confounder would need to be associated with at least a 1.39-fold increased risk for both the primary exposure and outcome, above and beyond the measured covariates.

## Discussion

Among women considered clinically eligible to plan VBAC without a history of psychotropic drug use in the year before birth, planned VBAC compared to ERCS was associated with a 15% reduced risk of the mother being dispensed any psychotropic medication and a 17% reduced risk of the mother being dispensed an antidepressant and at least two consecutive antidepressants in the first year postpartum. These findings were adjusted for many potential confounders as well as breastfeeding at 6–8 weeks postpartum, a potential mediating factor.

### Strengths and limitations of study

Strengths of this study include its large population-based design with very little loss to follow-up, which reduces the risk of selection bias and maximises statistical power. Other strengths include confining the study to women who were considered clinically eligible to plan a VBAC based on current UK guidelines, and the use of prospectively collected routine data which avoids recall bias. We were also able to explore the influence of many *a priori* potential confounders as well as the influence of breastfeeding, a potential mediating factor. However, similar to other observational studies, we cannot rule out residual confounding due to unmeasured confounding factors that we have not been able to consider such as indication for the previous caesarean. The reported *E*-values suggest that relatively modest unmeasured confounding could fully explain the observed associations. However, it is worth noting that in the absence of conducting a large randomised trial, which a previous study (Crowther, Dodd, Hiller, Haslam, & Robinson, 2012) indicates is unlikely to be feasible, large population-based observational studies with the ability to consider multiple covariates, such as our study, offer the best opportunity to inform evidence in this area.

Inferring treated mental health problems only from dispensed prescriptions of psychotropic medication is a limitation of our study. Our study is likely to have underestimated the proportion of women with treated mental health problems in the first year postpartum, as while psychotropic medication tends to be the most commonly used type of mental health treatment (McManus, Bebbington, Jenkins, & Brugha, 2016; Petersen, Peltola, Kaski, Walters, & Hardoon, 2018), other treatments, most notably psychological interventions, may be offered. There may also be a higher threshold in the postnatal period for using psychotropic medication amid concerns about the safety of these drugs for the breastfed baby (Tripathi & Majumder, 2010), noting, however, that our effects were still evident in women who did and did not breastfeed with no evidence that breastfeeding modified the effects of the exposures. We also did not have information about whether women actually took the dispensed medication or the indication for the medication. We also acknowledge that excluding women dispensed psychotropic drugs in the year, or 2 years in the case of the sensitivity analysis (online Supplementary Tables S9–S11), before they gave birth would have been unlikely to have excluded all women with previous mental health problems linked to the points raised above and the fact that not all women with mental health problems will present for treatment (McManus et al., 2016). Although the time when psychotropic drugs are most likely to be stopped is around the time of pregnancy recognition and restarting treatment in pregnancy is not uncommon (Petersen et al., 2016), we may also have not excluded all women with previous mental health problems as some women may have discontinued psychotropic drugs when trying to conceive. Furthermore, some women develop mental health problems *de novo* during pregnancy and remain untreated or are not treated with medication. However, this would only have biased the results if the proportion of women with either untreated or non-pharmacologically treated mental health problems before birth differed between those who planned a VBAC and those that had an ERCS.

We acknowledge the performance of multiple comparisons would have increased the risk of type 1 error, although using a more stringent *p* value to allow for multiple testing would not have altered our main findings. The amount of missing data on one or more of the covariates is recognised as another limitation. However, our use of multiple imputation is regarded as a valid method for dealing with this problem provided the unobserved data are missing at random and the models to impute the missing data have been correctly specified (Nguyen, Carlin, & Lee, 2017).

### Comparison with other studies and interpretation

To our knowledge, only one small study of under 300 women (Law et al., 2010) conducted in a single hospital in Hong Kong has to date investigated the effect of planned VBAC compared to ERCS on women's mental health. This prior study used psychometric tests during pregnancy and up to 6 months postpartum to assess anxiety, depression and general psychological wellbeing, reporting no significant differences between women who planned a VBAC compared to an ERCS. Consistent with our findings, another small study of 169 Australian women with one previous caesarean found that women who had a spontaneous vaginal birth were less likely than those giving birth by planned or emergency repeat caesarean or instrumental vaginal birth to have symptoms suggestive of postnatal depression (Shorten & Shorten, 2012). More recently, and again consistent with our findings, a study of nearly 900 women conducted in 15 maternity units in Europe reported that compared to women who had an emergency or planned repeat caesarean, women who had a spontaneous vaginal birth after one previous caesarean had higher postnatal health-related quality of life scores encompassing physical, psychological and social dimensions of health (Fobelets et al., 2018). A further analysis of the same study population found that women with an antenatal preference for vaginal birth who gave birth by ERCS had lower postnatal health-related quality of life scores compared to women with an antenatal preference for vaginal birth who actually gave birth vaginally (Fobelets et al., 2019). Our effect sizes were stronger in women who planned and actually had a VBAC than in women who planned but had an in-labour non-elective repeat caesarean, supporting the potential importance of a match between preferred and actual mode of birth on women's wellbeing.

Although more studies have been conducted in the general obstetric population, as mentioned in the Introduction, these studies may not be applicable to women with previous caesarean and most have focused on the effect of actual mode of birth on postnatal depression. Caesarean section has been reported to be both protective of and a risk factor for postnatal depression (Carter et al., 2006). A limited number of studies have also reported caesarean section to be a risk factor for postpartum psychosis (Kendell et al., 1987) and anxiety disorders such as post-traumatic stress disorder (Ayers et al., 2016). However, other studies have not found a significant association between mode of birth and these outcomes or have only found an association in women with a previous history of mental health problems (Carter et al., 2006; Valdimarsdottir et al., 2009). These inconsistencies in findings may partly reflect limitations with the existing studies, with many comprising small non-population-based studies that have not differentiated between planned and emergency caesarean despite the very different contexts in which these two types of caesarean are performed. Unplanned in contrast to planned caesareans are often carried out with little time for women to prepare themselves and are frequently accompanied by concerns around the safety of the mother and/or her baby. A recent meta-analysis of a small number of studies that did distinguish between type of caesarean, reported that unplanned but not planned caesareans were associated with an increased risk of postnatal depression (Xu, Ding, Ma, Xin, & Zhang, 2017).

Mode of birth may affect the mother's postnatal wellbeing by influencing the likelihood of the mother or baby experiencing birth-related complications. However, our study found that despite being more likely to experience a range of adverse perinatal and maternal intrapartum or postpartum outcomes (Fitzpatrick et al., 2019), women who planned a VBAC were less likely than those giving birth by ERCS to be dispensed psychotropic medication including antidepressants in the first year postpartum. This may be because women who planned a VBAC were less likely than those having an ERCS to seek treatment for mental health problems or were less likely to be offered and/or accept pharmacological treatment in the first year postpartum. Our findings may alternatively imply that women giving birth by ERCS are more likely than those having a planned VBAC to experience mental health problems severe enough to warrant pharmacological treatment, at least in the year after birth. This may arise through several potential mechanisms. Women giving birth by caesarean are more likely to express not feeling in control over their birth, may be more likely to have their expectations about childbirth violated, even in the case of planned caesarean and may experience a more painful and difficult postnatal recovery, all of which may adversely affect their subsequent mental health (Lobel & DeLuca, 2007). Caesarean birth may also adversely impact maternal mental health by disrupting physiological hormonal processes such as the release of oxytocin, a hormone which helps to reduce stress, increase sociability and maternal–infant attachment (Buckley, 2015). Although we excluded women with a history of psychotropic drug use in the 1–2 years before birth, we also cannot rule out that the possible increased likelihood of mental health problems in women giving birth by ERCS may be due to more prior psychiatric vulnerability in this group of women. Indeed, there is some evidence that women with a preference for elective caesarean are more likely to have a fear of childbirth, which in itself is thought to be associated with mental health problems (Saisto & Halmesmaki, 2003). It is also possible that women who plan VBAC may be more motivated and have personality traits associated with better mental health.

## Conclusions

This study found that among women considered clinically eligible to plan VBAC without a history of psychotropic drug use in the year before birth, planned VBAC compared to ERCS was associated with a reduced risk of the mother being dispensed psychotropic medication including antidepressants in the first year postpartum. Our findings should not be extrapolated to the general obstetric population, where the majority of caesareans in high-income countries are recommended for obstetric or medical reasons rather than reflective of women's choice. Rather, our findings are likely to be generalisable to women with previous caesarean section giving birth to a term singleton in other high-income countries with similar population characteristics and clinical practice. Further research is needed to establish if our findings reflect a causal increase in the likelihood of postnatal mental health problems in women giving birth by ERCS compared to planned VBAC or whether there are other reasons behind our findings, particularly as an increasing number of women in many countries face a choice about how to plan to give birth after previous caesarean.

## Supplementary material

For supplementary material accompanying this paper visit https://doi.org/10.1017/S0033291720005322. click here to view supplementary material

## References

[ref1] American College of Obstetricians and Gynecologists. (2019). ACOG practice bulletin no. 205: Vaginal birth after cesarean delivery. Obstetrics & Gynecology, 133(2), e110–e127. 30681543 10.1097/AOG.0000000000003078

[ref2] Ayers, S., Bond, R., Bertullies, S., & Wijma, K. (2016). The aetiology of post-traumatic stress following childbirth: A meta-analysis and theoretical framework. Psychological Medicine, 46(6), 1121–1134. doi: 10.1017/S0033291715002706 26878223

[ref3] Bartlett, J. W., Seaman, S. R., White, I. R., & Carpenter, J. R. (2014). Multiple imputation of covariates by fully conditional specification: Accommodating the substantive model. Statistical Methods in Medical Research, 24, 462–487. 24525487 10.1177/0962280214521348 PMC4513015

[ref4] Betran, A. P., Ye, J., Moller, A. B., Zhang, J., Gulmezoglu, A. M., & Torloni, M. R. (2016). The increasing trend in caesarean section rates: Global, regional and national estimates: 1990–2014. PLoS One, 11(2), e0148343. doi: 10.1371/journal.pone.0148343 26849801 PMC4743929

[ref5] Borra, C., Iacovou, M., & Sevilla, A. (2015). New evidence on breastfeeding and postpartum depression: The importance of understanding women's intentions. Maternal and Child Health Journal, 19(4), 897–907. doi: 10.1007/s10995-014-1591-z 25138629 PMC4353856

[ref6] Buckley, S. J. (2015). Executive summary of hormonal physiology of childbearing: Evidence and implications for women, babies, and maternity care. The Journal of Perinatal Education, 24(3), 145–153. doi: 10.1891/1058-1243.24.3.145 26834435 PMC4720867

[ref7] Carter, F. A., Frampton, C. M., & Mulder, R. T. (2006). Cesarean section and postpartum depression: A review of the evidence examining the link. Psychosomatic Medicine, 68(2), 321–330. doi: 10.1097/01.psy.0000204787.83768.0c 16554400

[ref8] Crowther, C. A., Dodd, J. M., Hiller, J. E., Haslam, R. R., & Robinson, J. S. (2012). Planned vaginal birth or elective repeat caesarean: Patient preference restricted cohort with nested randomised trial. PLoS Medicine, 9(3), e1001192. 22427749 10.1371/journal.pmed.1001192 PMC3302845

[ref9] Field, T. (2018). Postnatal anxiety prevalence, predictors and effects on development: A narrative review. Infant Behavior and Development, 51, 24–32. doi: 10.1016/j.infbeh.2018.02.005 29544195

[ref10] Fitzpatrick, K. E., Kurinczuk, J. J., Alfirevic, Z., Spark, P., Brocklehurst, P., & Knight, M. (2012). Uterine rupture by intended mode of delivery in the UK: A national case-control study. PLoS Medicine, 9(3), e1001184. 22427745 10.1371/journal.pmed.1001184 PMC3302846

[ref11] Fitzpatrick, K. E., Kurinczuk, J. J., Bhattacharya, S., & Quigley, M. A. (2019). Planned mode of delivery after previous cesarean section and short-term maternal and perinatal outcomes: A population-based record linkage cohort study in Scotland. PLoS Medicine, 16(9), e1002913. doi: 10.1371/journal.pmed.1002913 31550245 PMC6759152

[ref12] Fobelets, M., Beeckman, K., Buyl, R., Daly, D., Sinclair, M., Healy, P., … Putman, K. (2018). Mode of birth and postnatal health-related quality of life after one previous cesarean in three European countries. Birth (Berkeley, Calif ), 45(2), 137–147. doi: 10.1111/birt.12324 29205463

[ref13] Fobelets, M., Beeckman, K., Buyl, R., Healy, P., Grylka-Baeschlin, S., Nicoletti, J., … Putman, K. (2019). Preference of birth mode and postnatal health related quality of life after one previous caesarean section in three European countries. Midwifery, 79, 102536. doi: 10.1016/j.midw.2019.102536 31561129

[ref14] Ghaedrahmati, M., Kazemi, A., Kheirabadi, G., Ebrahimi, A., & Bahrami, M. (2017). Postpartum depression risk factors: A narrative review. Journal of Education and Health Promotion, 6, 60. doi: 10.4103/jehp.jehp_9_16 28852652 PMC5561681

[ref15] Gregory, K. D., Korst, L. M., Fridman, M., Shihady, I., Broussard, P., Fink, A., & Burnes Bolton, L. (2008). Vaginal birth after cesarean: Clinical risk factors associated with adverse outcome. American Journal of Obstetrics and Gynecology, 198(4), 452 e451–410; discussion 452, e410–452. doi:10.1016/j.ajog.2008.01.008. 18395037

[ref16] Guise, J.-M., Eden, K., Emeis, C., Denman, M. A., Marshall, N., Fu, R. R., … McDonagh, M. (2010). Vaginal birth after cesarean: New insights. Evidence Report/Technology Assessment (191), 1–397. PMC4781304 20629481

[ref17] Information Services Division **Scotland**. (2019). Births in Scottish hospitals year ending 31 March 2019. Retrieved from https://www.isdscotland.org/Health-Topics/Maternity-and-Births/Publications/2019-11-26/2019-11-26-Births-Report.pdf. Accessed 7 October 2020

[ref18] Information Services Division Scotland. (2018). Medicines used in mental health – BNF Legacy. Retrieved from https://www.isdscotland.org/Health-Topics/Prescribing-and-medicines/Community-Dispensing/Mental-Health/. Accessed 25 November 2019.

[ref19] Kendell, R. E., Chalmers, J. C., & Platz, C. (1987). Epidemiology of puerperal psychoses. British Journal of Psychiatry, 150, 662–673. doi: 10.1192/bjp.150.5.662 3651704

[ref20] Law, L. W., Pang, M. W., Chung, T. K.-H., Lao, T. T.-H., Lee, D. T.-S., Leung, T. Y., … Lau, T. K. (2010). Randomised trial of assigned mode of delivery after a previous cesarean section – impact on maternal psychological dynamics. The Journal of Maternal-Fetal & Neonatal Medicine, 23(10), 1106–1113. 20088723 10.3109/14767050903551434

[ref21] Lobel, M., & DeLuca, R. S. (2007). Psychosocial sequelae of cesarean delivery: Review and analysis of their causes and implications. Social Science & Medicine, 64(11), 2272–2284. doi: 10.1016/j.socscimed.2007.02.028. 17395349

[ref22] McManus, S., Bebbington, P., Jenkins, R., & Brugha, T. (Eds.) (2016). Mental health and wellbeing in England: Adult psychiatric morbidity survey 2014. Leeds: NHS Digital.

[ref23] National Institute for Health and Clinical Excellence. (2011). Caesarean section NICE clinical guideline 132. Retrieved from https://www.nice.org.uk/guidance/cg132/resources/caesarean-section-pdf-35109507009733. Accessed 8 August 2018. 31886991

[ref24] Nguyen, C. D., Carlin, J. B., & Lee, K. J. (2017). Model checking in multiple imputation: An overview and case study. Emerging Themes in Epidemiology, 14, 8. doi: 10.1186/s12982-017-0062-6 28852415 PMC5569512

[ref25] NHS Digital. (2019). NHS maternity statistics, England 2018–19. Retrieved from https://digital.nhs.uk/data-and-information/publications/statistical/nhs-maternity-statistics/2018-19. Accessed 7 October 2020

[ref26] O'Hara, M. W., & McCabe, J. E. (2013). Postpartum depression: Current status and future directions. Annual Review of Clinical Psychology, 9, 379–407. doi: 10.1146/annurev-clinpsy-050212-185612 23394227

[ref27] Petersen, I., McCrea, R. L., Sammon, C. J., Osborn, D. P., Evans, S. J., Cowen, P. J., … Nazareth, I. (2016). Risks and benefits of psychotropic medication in pregnancy: Cohort studies based on UK electronic primary care health records. Health Technology Assessment, 20(23), 1–176. doi: 10.3310/hta20230 PMC4827034 27029490

[ref28] Petersen, I., Peltola, T., Kaski, S., Walters, K. R., & Hardoon, S. (2018). Depression, depressive symptoms and treatments in women who have recently given birth: UK cohort study. BMJ Open, 8(10), e022152. doi: 10.1136/bmjopen-2018-022152 PMC6224756 30361401

[ref29] The Royal Australian and New Zealand College of Obstetricians and Gynaecologists. (2015). Birth after previous caesarean section. Retrieved from https://ranzcog.edu.au/RANZCOG_SITE/media/RANZCOG-MEDIA/Women%27s%20Health/Statement%20and%20guidelines/Clinical-Obstetrics/Birth-after-previous-Caesarean-Section-(C-Obs-38)Review-March-2019.pdf?ext=.pdf. Accessed 10 April 2019.

[ref30] Royal College of Obstetricians and Gynaecologists. (2010). Late intrauterine fetal death and stillbirth green-top guidelines no. 55. Retrieved from https://www.rcog.org.uk/globalassets/documents/guidelines/gtg_55.pdf. Accessed 16 May 2018.

[ref31] Royal College of Obstetricians and Gynaecologists. (2015). Birth after previous caesarean birth, green-top guideline no. 45. Retrieved from https://www.rcog.org.uk/globalassets/documents/guidelines/gtg_45.pdf. Accessed 8 August 2017.

[ref32] Saisto, T., & Halmesmaki, E. (2003). Fear of childbirth: A neglected dilemma. Acta Obstetricia et Gynecologica Scandinavica, 82(3), 201–208. 12694113

[ref33] Shorten, A., & Shorten, B. (2012). The importance of mode of birth after previous cesarean: Success, satisfaction, and postnatal health. Journal of Midwifery & Women's Health, 57(2), 126–132. doi: 10.1111/j.1542-2011.2011.00106.x 22432483

[ref34] Slomian, J., Honvo, G., Emonts, P., Reginster, J. Y., & Bruyere, O. (2019). Consequences of maternal postpartum depression: A systematic review of maternal and infant outcomes. Women's Health *(*Lond*)*, 15, 1745506519844044. doi: 10.1177/1745506519844044 31035856 PMC6492376

[ref35] Tripathi, B. M., & Majumder, P. (2010). Lactating mother and psychotropic drugs. Mens Sana Monographs, 8(1), 83–95. doi: 10.4103/0973-1229.58821 21327172 PMC3031938

[ref36] Valdimarsdottir, U., Hultman, C. M., Harlow, B., Cnattingius, S., & Sparen, P. (2009). Psychotic illness in first-time mothers with no previous psychiatric hospitalizations: A population-based study. PLoS Medicine, 6(2), e13. doi: 10.1371/journal.pmed.1000013 19209952 PMC2637917

[ref37] VanderWeele, T. J., & Ding, P. (2017). Sensitivity analysis in observational research: Introducing the *E*-value. Annals of Internal Medicine, 167(4), 268–274. doi: 10.7326/M16-2607 28693043

[ref38] Vittinghoff, E., & McCulloch, C. E. (2007). Relaxing the rule of ten events per variable in logistic and Cox regression. American Journal of Epidemiology, 165(6), 710–718. 17182981 10.1093/aje/kwk052

[ref39] Welsh Government. (2020). Maternity and birth statistics, Wales 2019. Retrieved from https://gov.wales/sites/default/files/statistics-and-research/2020-08/maternity-and-birth-statistics-2019-updated.pdf. Accessed 7 October 2020

[ref40] White, I. R., Royston, P., & Wood, A. M. (2009). Multiple imputation using chained equations: Issues and guidance for practice. Statistics in Medicine, 30, 377–399. 10.1002/sim.4067 21225900

[ref41] Xu, H., Ding, Y., Ma, Y., Xin, X., & Zhang, D. (2017). Cesarean section and risk of postpartum depression: A meta-analysis. Journal of Psychosomatic Research, 97, 118–126. doi: 10.1016/j.jpsychores.2017.04.016. 28606491

