# Supplementary material for: Planned mode of birth after previous caesarean section and women's use of psychotropic medication in the first year postpartum: a population-based record linkage cohort study
Source: Psychol Med. 2021 Jan 28;52(14):3210–21. doi: 10.1017/S0033291720005322 (PMC9693703; doi:10.1017/S0033291720005322)
Supplement: Supplementary file 1 [file S0033291720005322sup.zip › S0033291720005322sup001.docx]

**Table S1. Data sources**

| **Data source** | **Description** |
| --- | --- |
| National Records of  Scotland (NRS) live births and stillbirths | Statutory data on all live births and stillbirths occurring in Scotland, subject to various quality checks (National Records of Scotland). |
| The Scottish Morbidity Record Maternity Inpatient and Day Case dataset (SMR02) | Inpatient and day case discharge data from all obstetric specialties in the National Health Service (NHS) Scotland. SMR02 has had a national coverage of around 98% of all births in NRS since the late 1970s (Information Services Division Scotland, 2017a), and is subject to regular quality checks (Information Services Division Scotland, 2010, 2019). The quality assurance exercises published in 2010 and 2019 found that the key fields used in this study, including mode of birth, matched the information found in the medical records in around 90% or more of the records sampled. |
| The Scottish Morbidity Record General/Acute Inpatient and Day case dataset (SMR01) | Inpatient and day case discharge data from all acute specialties in NHS Scotland, subject to regular quality checks (Information Services Division Scotland, 2012). |
| The Prescribing Information System (PIS) | Data on all prescriptions dispensed in the community in Scotland, subject to regular quality checks demonstrating consistent accuracy of ≥98% (Information Services Division Scotland, 2016). Data was available from 2009-2016 at the time it was obtained. |
| NRS deaths | Statutory data about all deaths occurring in Scotland, subject to various quality checks (National Records of Scotland). |
| The Child Health Surveillance Programme Pre-School system (CHSP-PS) | Data collected during health reviews carried out on pre-school children in Scotland including infant feeding information. The number of Health Boards in Scotland using CHSP-PS has increased over time, with the quality of information recorded on infant feeding reported to be high (Information Services Division Scotland, 2017b). |
| The Community Health Index (CHI) database | A register of all individuals in NHS Scotland. This was used to determine dates of immigration to or emigration from Scotland. |

**Table S2. Data sources, codes and database fields used to identify study population, exposures, outcomes and covariates**

|  | **Data source** | **Database fields/codes** |
| --- | --- | --- |
| **Inclusion criteria** |  |  |
| ≥ 1 previous caesarean sections | SMR02 | *Previous caesarean sections* field ≥1 OR at least one previous delivery with a code for caesarean section in *mode of delivery* field (7 or 8) and/or an OPCS-4 or OPCS-3 code for caesarean section (R17-R18, R251, 764-766, 769), using SMR02 records going back as far as 1981 |
| Singleton birth | NRS live births and stillbirths | *Numbirths* field=1 |
| Term birth | SMR02 | Gestation at delivery 37-41 completed weeks according to *Estimated gestation* field (containing number of completed weeks of gestation as judged by the clinician, usually on the basis of ultrasound) OR according to gestation derived from *date of delivery* and *date of last menstrual period* fields if *Estimated gestation* missing (0.06% of eligible births) |
| **Exclusion criteria** |  |  |
| Non-cephalic presentation | SMR02 | Delivery episodes with a code for breech or shoulder in *presentation at delivery* field (4 or 6) OR a code for breech delivery or breech extraction in *mode of delivery* field (5 or 6) OR an ICD-10 code for breech delivery or breech extraction (O801, O830-O831) OR an OPCS-4 code for breech delivery or breech extraction (R19-R20) OR an ICD-10 code for maternal care for malpresentation of fetus (0320-0322, 0326-0329) |
| Placenta praevia | SMR02 | Delivery episodes with an ICD-10 code for placenta praevia (O440-O441) |
| Abdominal pregnancy | SMR02 | Delivery episodes with an ICD-10 code for delivery of or maternal care for viable fetus in abdominal pregnancy (O833, O367) |
| Known or suspected disproportion of maternal and/or fetal origin | SMR02 | Delivery episodes with an ICD-10 code for maternal care for known or suspected disproportion (O33) |
| Tumour of corpus uteri | SMR02 | Delivery episodes with an ICD-10 code for maternal care for tumour of corpus uteri (O341) |
| Pre-labour non-elective caesarean section | SMR02 | Code for non-elective caesarean section in *mode of delivery* field (8) AND *duration of labour* field=0 |
| Antepartum stillbirth | NRS stillbirths | Code for antepartum stillbirth in *period of death* field (1) |
| Migrated to Scotland < 1 year before birth | CHI database and SMR02 | Derived from *date of immigration* and *date of delivery* fields |
| **Exposures** |  |  |
| Elective repeat caesarean section (ERCS) | SMR02 | Code for elective caesarean section in *mode of delivery* field (7) in women with ≥ 1 previous caesarean sections |
| Planned vaginal birth after previous caesarean section (planned VBAC) | SMR02 | Code for vaginal delivery in *mode of delivery* field (0, 1, 2, 3, 4, A, B, C, D or E) OR code for non-elective caesarean section in *mode of delivery* field (8) AND *duration of labour* field ≥1 hour in women with ≥1 previous caesarean sections |
| Planned VBAC without labour induction | SMR02 | Criteria for Planned VBAC AND code for none in *induction of labour* field (0) |
| Planned VBAC with labour induction | SMR02 | Criteria for Planned VBAC AND code for induction of labour using artificial rupture of membranes (ARM), oxytocics, ARM & oxytocics, prostaglandins, prostaglandins & ARM, prostaglandins & oxytocics, prostaglandins & ARM & oxytocics or other method in *induction of labour* field (1-8). Surgical induction defined as using ARM to induce labour and medical induction defined as using oxytocics and/or prostaglandins to induce labour. |
| Vaginal birth after previous caesarean section (VBAC) | SMR02 | Code for vaginal delivery in *mode of delivery* field (0, 1, 2, 3, 4, A, B, C, D or E) in women with ≥ 1 previous caesarean sections |
| In-labour non-elective repeat caesarean section | SMR02 | Code for non-elective caesarean section in *mode of delivery* field (8) AND *duration of labour* field ≥1 hour in women with ≥1 previous caesarean sections |
| **Outcomes** |  |  |
| Dispensed any psychotropic medication | PIS | First dispensed date within 12 months of delivery for any medications in the ‘legacy’ British National Formulary (BNF)(Information Services Division Scotland, 2018) subsection 40101 (Hypnotics) or 40102 (Anxiolytics), or BNF section code 402 (Drugs used in psychoses and related disorders) or BNF subsection codes 40301 (Tricyclic and related antidepressant drugs), 40302 (Monoamine-oxidase inhibitors), 40303 (Selective serotonin re-uptake inhibitor) or 40304 (Other antidepressant drugs), excluding BNF item description “Amitriptlyine Hcl Tab 10mg” |
| Dispensed an antidepressant | PIS | First dispensed date within 12 months of delivery for any medications in the BNF subsection 40301 (Tricyclic and related antidepressant drugs), 40302 (Monoamine-oxidase inhibitors), 40303 (Selective serotonin re-uptake inhibitor) or 40304 (Other antidepressant drugs), excluding BNF item description “Amitriptlyine Hcl Tab 10mg” |
| Dispensed at least two consecutive prescriptions for an antidepressant | PIS | First dispensed date within 12 months of delivery for any medications in the BNF subsection 40301 (Tricyclic and related antidepressant drugs), 40302 (Monoamine-oxidase inhibitors), 40303 (Selective serotonin re-uptake inhibitor) or 40304 (Other antidepressant drugs), that was followed by at least one consecutive prescription for any medications in the BNF subsection 40301, 40302, 40303 or 40304, not including BNF item description “Amitriptlyine Hcl Tab 10mg”. Prescriptions were considered to be consecutive if the second prescription was dispensed within 91 days of the first dispensed prescription |
| Dispensed a hypnotic and/or anxiolytic | PIS | First dispensed date within 12 months of delivery for any medications in the BNF subsection 40101 (Hypnotics) or 40102 (Anxiolytics) |
| Dispensed a antipsychotic and/or related drug | PIS | First dispensed date within 12 months of delivery for any medications in the BNF section 402 (Drugs used in psychoses and related disorders) |
| **Socio-demographic characteristics** |  |  |
| Maternal age | SMR02 | Derived from mother’s *date of birth* and *date of delivery* in current pregnancy |
| Mother’s country of birth | NRS live and stillbirths | *Mother’s Country of Birth* field |
| Marital status/registration type | NRS live and stillbirths | *Parents married indicator* field |
| Socio-economic status | NRS live and stillbirths | Socio-economic status of mother if sole registered birth or highest of mother or father’s socio-economic status for births registered inside marriage or jointly registered by both parents outside marriage. Socio-economic status defined by National Statistics Socio-Economic Classification (NS-SEC) based on occupation and employment status |
| **Maternal medical and pregnancy-related characteristics** |  |  |
| Number of pervious caesarean sections | SMR02 | *Previous caesarean sections* field. Number of previous caesarean sections according to previous caesarean sections field was cross-checked against woman’s previous delivery records in SMR02. Where this was found to be less than the number of previous caesarean sections observed to date (15% of eligible births), it was overwritten with the higher number. Also, if the previous caesarean sections field was missing (0.2% of eligible births), number of previous caesarean sections was derived from the number of previous caesarean sections observed to date. |
| Any prior vaginal birth | SMR02 | Derived from parity (number of previous pregnancies resulting in either a live birth or stillbirth) and number of previous caesarean sections. Woman’s previous delivery records were also examined for evidence of any prior vaginal deliveries. |
| Inter-pregnancy interval | SMR02 | Derived from interval between *date of delivery* of current pregnancy and *date of delivery* of previous delivery minus gestational age at delivery of current pregnancy |
| Any previous stillbirth or neonatal death | SMR02 | Derived from *previous stillbirths* and *previous neonatal deaths* fields. |
| Maternal smoking status at booking | SMR02 | *Booking smoking history* field |
| Maternal BMI at booking | SMR02 | Derived from *height* and *weight of mother at booking* fields. Maternal height values < 120 cm and > 200 cm, weight of mother at booking values < 32kg and > 180 kg and maternal BMI at booking values ≤15 and >80 kg/m2 were considered to be implausible based on published values (Information Services Division Scotland; Knight, Kurinczuk, Spark, Brocklehurst, & UKOSS, 2010). If height was missing or implausible, it was set to the median value observed in the woman’s other records in SMR02. Implausible values of maternal weight or BMI at booking were set to missing. |
| Any hypertensive disorder | SMR02 | Antenatal or delivery episodes with an ICD-10 code for pre-existing or gestational hypertensive disorder (O10-O11, O13-O16, I10) |
| Pre-existing or gestational diabetes mellitus | SMR02 | Antenatal or delivery episodes with a code for pre-existing or gestational diabetes mellitus in *diabetes* field (1-3) OR an ICD-10 code for pre-existing or gestational diabetes mellitus (O24, E10-E11) |
| **Intrapartum and postpartum characteristics** |  |  |
| Adverse perinatal outcome (Including intrapartum stillbirth or neonatal death, admission to a neonatal unit, resuscitation requiring drugs and/or intubation or an Apgar score < 7 at 5 minutes) | NRS deaths & SMR02 | Code for intrapartum stillbirth or death within four weeks of birth in *period of death* field (2-6), code for admitted in *neonatal indicator* field (1-2), code for bag and mask with drugs, intubation for IPVV with/without drugs or drugs only in *resuscitation* field (3-6), or < 7 in *Apgar score* field |
| Maternal intrapartum or postpartum complication (including uterine rupture, peripartum hysterectomy, blood transfusion, puerperal sepsis, other puerperal infection, surgical injury – damage to bowel, bladder or ureter requiring surgical repair, third- or fourth-degree perineal tear, or overnight readmission to hospital within 42 days of birth) | SMR02 and SMR01 | Delivery episodes with an ICD-10 code for uterine rupture (O710-O711); OPCS-4 code for hysterectomy (Q071-Q075, Q08, R251) within 6 weeks of delivery; Delivery episodes with an OPCS-4 code for blood transfusion (X331-X333, X337-X339, X341); ICD-10 code for puerperal sepsis (O85) within 6 weeks of delivery; ICD-10 code for other puerperal infections (O86) within 6 weeks of delivery; OPCS-4 code for any of the following within 6 weeks of delivery: total excision of colon and rectum, total excision of colon, extended excision of right hemicolon, other excision of right hemicolon, excision of transverse colon, excision of left hemicolon, excision of sigmoid colon, other excision of colon, exteriorisation of caecum, other exteriorisation of colon, subtotal excision of colon, exteriorisation of colon, repair of anus, other operations on the anal sphincter to control continence, excision of ureter, urinary diversion, replantation of ureter, other connection of ureter, repair of ureter, incision of ureter, other open operations on ureter,therapeutic nephroscopic operations on ureter, therapeutic ureteroscopic operations on ureter, other therapeutic endoscopic operations on ureter, percutaneous ureteric stent procedures, total excision of bladder, partial excision of bladder, enlargement of bladder, other repair of bladder or open drainage of bladder (H04-H11, H14-H15, H29, H32, H50, H57, M18-M23, M25-M27, M29, M33-M38); Delivery episodes with a code for third- or fourth-degree tear in *tears* field (3 or 4) OR ICD-10 code for third- or fourth-degree perineal laceration during delivery (O702-0703) OR OPCS-4 code for repair of obstetric laceration of perineum and sphincter of anus or repair of obstetric laceration of perineum and sphincter and mucosa of anus (R322, R325); or women readmitted to hospital within 42 days of delivery, excluding planned transfers and readmissions of less than one day. Planned transfers identified as episodes where the difference between the admission date of the episode and the discharge date of the previous episode is less than 0 OR is between 0-1 day AND either the admission from/type of admission is a transfer or the discharged to/type of discharge of the previous episode is a transfer. Maternal deaths within the delivery episode were excluded, identified using discharge type 40-43 (death). Women with a length of postnatal hospital stay >42 days were also excluded. |
| Any breastfeeding at 6-8 week review | CHSP-PS | Method of feeding at 6-8 week review breast milk only or mixed breast and formula milk |

**
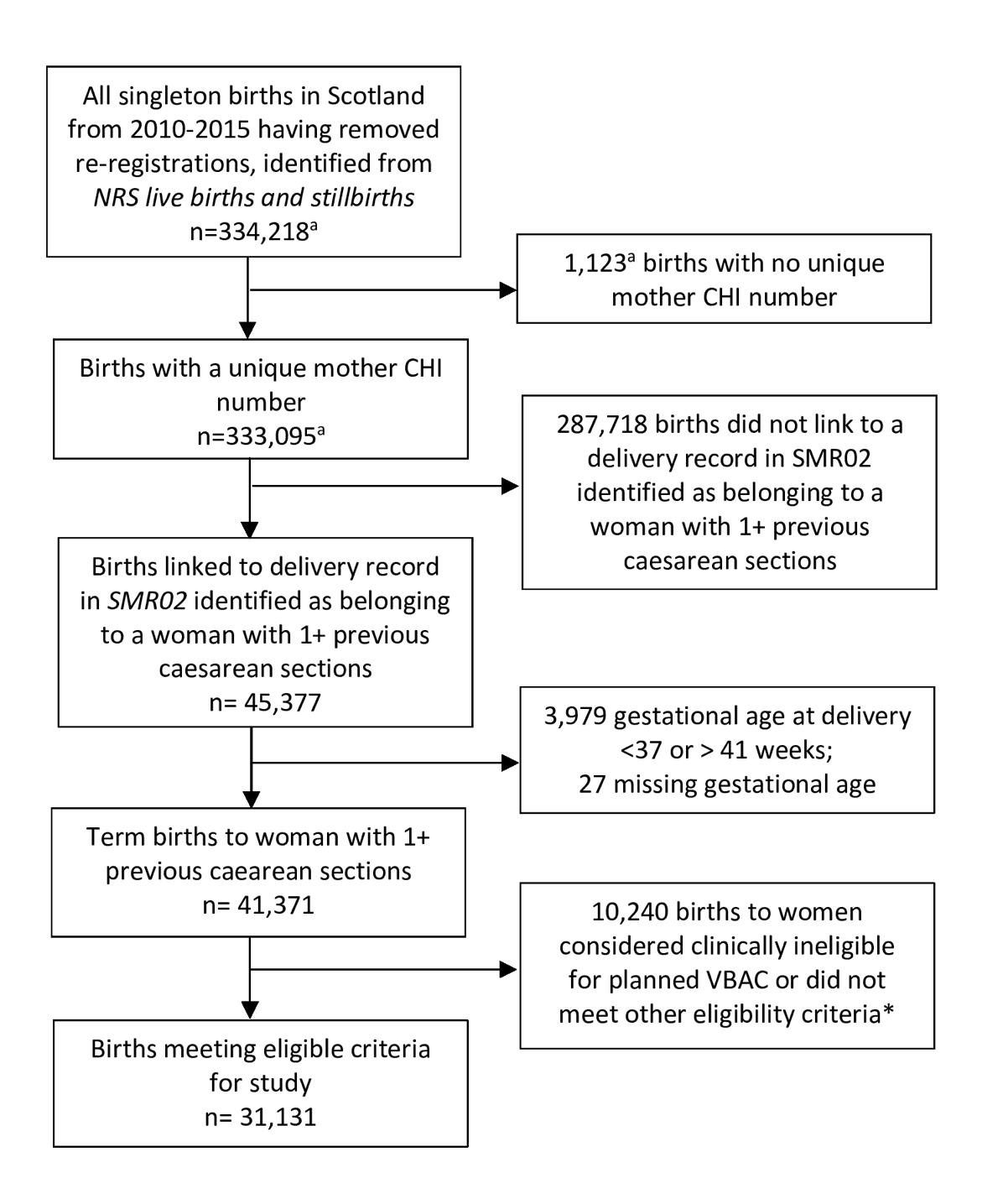
**

**Figure S1. Flow diagram of cohort selection**

*Clinically ineligible for planned VBAC or did not meet other eligibility criteria for study because of one or more of the following: non-cephalic presentation at birth (n=2,214); placenta praevia (n=289); abdominal pregnancy (n=1); known or suspected disproportion of maternal and/or fetal origin (n=23); tumour of corpus uteri (n=150); birth by pre-labour non-elective caesarean section (n=2,279); antepartum stillbirth (n=54); dispensed any of the primary outcome medications in the year before birth (n=5,006); migrated to Scotland less than one year before birth (n=234); stillbirth missing time of death in relation to birth (n=1); missing information on mode of birth (n=3); birth by non-elective caesarean section missing information about duration of labour (n=984); and number of previous caesarean sections greater than parity (n=145). Reasons not mutually exclusive.

^a^Numbers provided by Information Services Division Scotland

Abbreviations: CHI, community health index; NRS, National Records of Scotland; SMR02, Scottish Morbidity Record Maternity Inpatient and Day Case dataset; VBAC, vaginal birth after previous caesarean


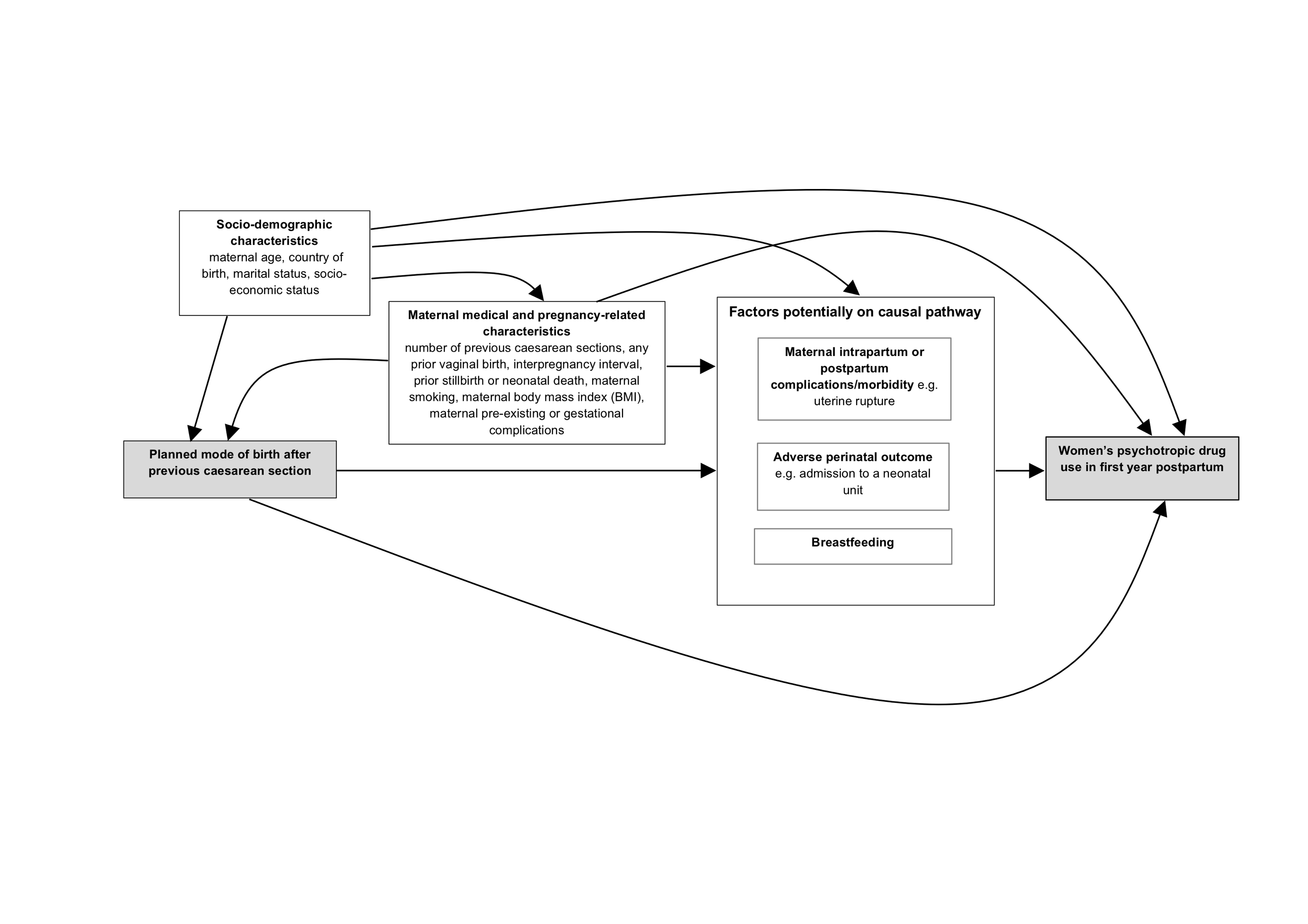


Figure S2. Conceptual framework of how socio-demographic, maternal medical and pregnancy-related factors might influence the relationship between planned mode of birth after previous caesarean and women’s psychtropic drug use in the first year postpartum

**
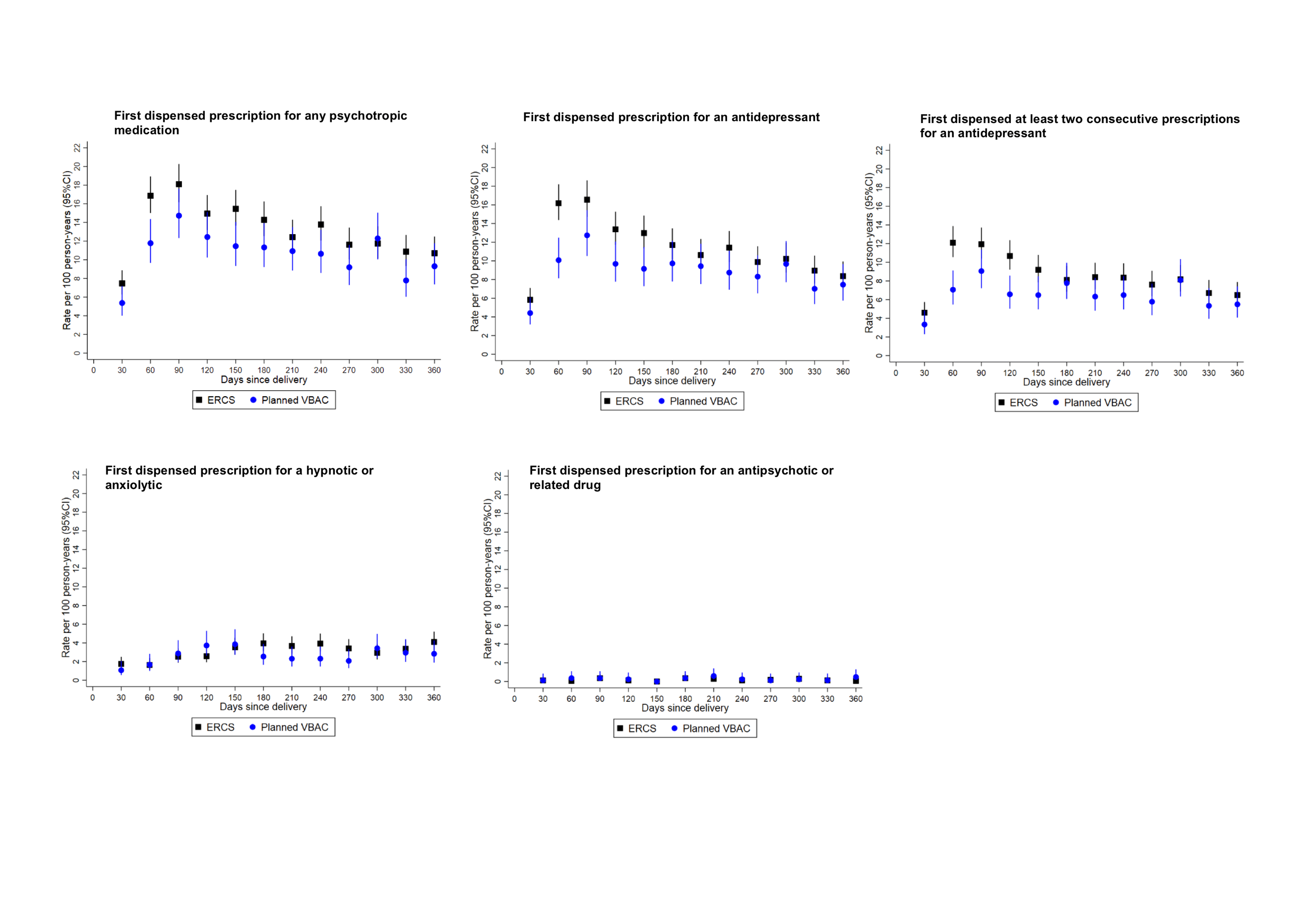
**

**Figure S3. Crude rates of each outcome over the first year postpartum**

Abbreviations: CI, confidence interval; ERCS, elective repeat caesarean section; VBAC, vaginal birth after previous caesarean

**
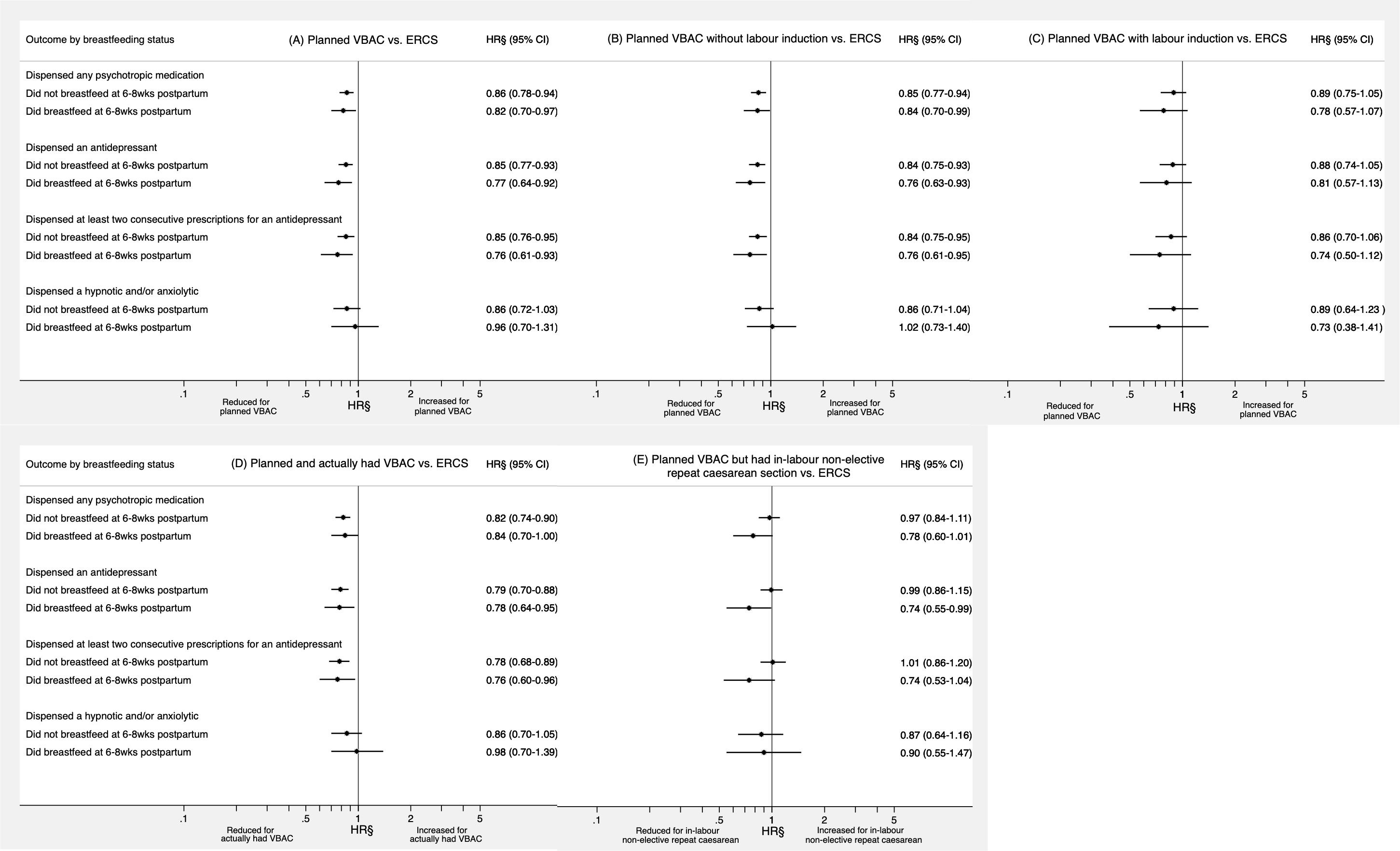
**

**Figure S4.** **Outcomes following (A) planned VBAC compared to ERCS, (B) planned VBAC without labour induction compared to ERCS, (C) planned VBAC with labour induction compared to ERCS, (D) planned and actually had VBAC compared to ERCS, (E) planned VBAC but had in-labour non-elective repeat caesarean section compared to ERCS by breastfeeding status at 6-8 weeks postpartum**

§adjusted for year of delivery, socio-demographic factors (maternal age, mother's country of birth, marital status and socio-economic status) and maternal medical and pregnancy-related factors (number of previous caesarean sections, any prior vaginal birth, inter-pregnancy interval, any prior stillbirth or neonatal death, maternal smoking status at booking, maternal BMI at booking, hypertensive disorder, and diabetes). Not shown for outcome antipsychotic and/or related drug because of low number of events.

P-value for interaction between planned mode of birth and breastfeeding at 6-8 weeks postpartum 0.643 for any psychotropic medication, 0.355 for an antidepressant outcomes, 0.346 for at least two consecutive prescriptions for an antidepressant, and 0.563 for a hypnotic and/or anxiolytic; p-value for interaction between planned mode of birth differentiated by whether labour was induced and breastfeeding at 6-8 weeks postpartum 0.767 for any psychotropic medication, 0.672 for an antidepressant, 0.641 for at least two consecutive prescriptions for an antidepressant, and 0.550 for a hypnotic and/or anxiolytic; p-value for interaction between actual mode of birth and breastfeeding at 6-8 weeks postpartum 0.312 for any psychotropic medication, 0.213 for an antidepressant, 0.266 for at least two consecutive prescriptions for an antidepressant, and 0.803 for a hypnotic and/or anxiolytic. Abbreviations: ERCS, elective repeat caesarean section; HR, Hazard ratio; VBAC, vaginal birth after previous caesarean

**Table S3. Complete case analysis of outcomes following planned VBAC compared to ERCS**

| **Outcomes** | **Base model^1^ HR (95% CI)** | **Model A^2^ HR (95% CI)** | **Model B^3^ HR (95% CI)** | **Model C^4^ HR (95% CI)** |
| --- | --- | --- | --- | --- |
| Dispensed any psychotropic medication | **0.80 (0.75 - 0.87)** | **0.80 (0.75 - 0.86)** | **0.85 (0.78 - 0.93)** | **0.88 (0.80 - 0.97)** |
|  | **P<0.001** | **P<0.001** | **P<0.001** | **P=0.008** |
| Dispensed an antidepressant | **0.78 (0.72 - 0.85)** | **0.78 (0.72 - 0.84)** | **0.82 (0.74 - 0.90)** | **0.86 (0.78 - 0.95)** |
|  | **P<0.001** | **P<0.001** | **P<0.001** | **P=0.003** |
| Dispensed at least two consecutive prescriptions for an antidepressant | **0.76 (0.69 - 0.83)** | **0.76 (0.69 - 0.83)** | **0.82 (0.74 - 0.92)** | **0.88 (0.79 - 0.99)** |
|  | **P<0.001** | **P<0.001** | **P<0.001** | **P=0.034** |
| Dispensed a hypnotic and/or anxiolytic | **0.85 (0.74 - 0.99)** | 0.87 (0.75 - 1.00) | 0.95 (0.80 - 1.12) | 0.96 (0.80 - 1.15) |
|  | **P=0.032** | P=0.054 | P=0.517 | P=0.688 |
| Dispensed an antipsychotic and/or related drug | 1.54 (0.94 - 2.52) | 1.50 (0.90 - 2.49) | NC | NC |
|  | P=0.088 | P=0.116 |  |  |

1 Base model adjusted for year of delivery only.

2 Model A adjusted for year of delivery and socio-demographic factors (maternal age, mother's country of birth, marital status and socio-economic status).

3 Model B adjusted for variables in Model A and additionally adjusted for maternal medical and pregnancy-related factors (number of previous caesarean sections, any prior vaginal birth, inter-pregnancy interval, any prior stillbirth or neonatal death, maternal smoking status at booking, maternal BMI at booking, hypertensive disorder, and diabetes).

4 Model C adjusted for variables in Model B and additionally adjusted for any breastfeeding at 6-8 weeks postpartum.

Bold text indicates statistically significant findings at the 5% level.

Abbreviations: CI, confidence interval; ERCS, elective repeat caesarean section; HR, Hazard ratio; NC, not calculated because of low number of events; VBAC, vaginal birth after previous caesarean

**Table S4. Complete case analysis of outcomes following planned VBAC with and without labour induction compared to ERCS**

|  | **Planned VBAC without labour induction vs. ERCS** | | | |  | **Planned VBAC with labour induction vs. ERCS** | | | |
| --- | --- | --- | --- | --- | --- | --- | --- | --- | --- |
|  | **Base model^1^ HR (95% CI)** | **Model A^2^ HR (95% CI)** | **Model B^3^ HR (95% CI)** | **Model C^4^ HR (95% CI)** |  | **Base model^1^ HR (95% CI)** | **Model A^2^ HR (95% CI)** | **Model B^3^ HR (95% CI)** | **Model C^4^ HR (95% CI)** |
| Dispensed any psychotropic medication | **0.79 (0.73 - 0.85)** | **0.80 (0.73 - 0.86)** | **0.84 (0.77 - 0.93)** | **0.88 (0.80 - 0.97)** |  | 0.87 (0.76 - 1.01) | **0.83 (0.72 - 0.96)** | 0.88 (0.75 - 1.04) | 0.90 (0.76 - 1.08) |
|  | **P<0.001** | **P<0.001** | **P<0.001** | **P=0.010** |  | P=0.060 | **P=0.012** | P=0.139 | P=0.261 |
| Dispensed an antidepressant | **0.76 (0.70 - 0.83)** | **0.76 (0.70 - 0.83)** | **0.81 (0.73 - 0.90)** | **0.85 (0.77 - 0.95)** |  | 0.87 (0.74 - 1.01) | **0.82 (0.71 - 0.96)** | 0.86 (0.71 - 1.03) | 0.88 (0.72 - 1.07) |
|  | **P<0.001** | **P<0.001** | **P<0.001** | **P=0.003** |  | P=0.065 | **P=0.011** | P=0.094 | P=0.188 |
| Dispensed at least two consecutive prescriptions for an antidepressant | **0.74 (0.67 - 0.82)** | **0.75 (0.68 - 0.83)** | **0.82 (0.73 - 0.93)** | 0.89 (0.79 - 1.01) |  | **0.82 (0.68 - 0.98)** | **0.78 (0.66 - 0.94)** | 0.82 (0.66 - 1.01) | 0.85 (0.67 - 1.07) |
|  | **P<0.001** | **P<0.001** | **P=0.001** | P=0.065 |  | **P=0.026** | **P=0.008** | P=0.066 | P=0.157 |
| Dispensed a hypnotic and/or anxiolytic | **0.85 (0.73 - 0.99)** | 0.87 (0.75 - 1.02) | 0.94 (0.78 - 1.13) | 0.95 (0.78 - 1.15) |  | 0.89 (0.67 - 1.17) | 0.87 (0.66 - 1.15) | 0.98 (0.71 - 1.35) | 1.04 (0.74 - 1.46) |
|  | **P=0.042** | P=0.088 | P=0.517 | P=0.606 |  | P=0.405 | P=0.332 | P=0.912 | P=0.816 |
| Dispensed an antipsychotic and/or related drug | 1.56 (0.92 - 2.64) | 1.54 (0.90 - 2.63) | NC | NC |  | 1.48 (0.58 - 3.78) | 1.39 (0.54 - 3.58) | NC | NC |
|  | P=0.098 | P=0.117 |  |  |  | P=0.412 | P=0.493 |  |  |

1 Base model adjusted for year of delivery only.

2 Model A adjusted for year of delivery and socio-demographic factors (maternal age, mother's country of birth, marital status and socio-economic status).

3 Model B adjusted for variables in Model A and additionally adjusted for maternal medical and pregnancy-related factors (number of previous caesarean sections, any prior vaginal birth, inter-pregnancy interval, any prior stillbirth or neonatal death, maternal smoking status at booking, maternal BMI at booking, hypertensive disorder, and diabetes).

4 Model C, adjusted for variables in Model B and additionally adjusted for any breastfeeding at 6-8 weeks postpartum.

Bold text indicates statistically significant findings at the 5% level.

Abbreviations: CI, confidence interval; ERCS, elective repeat caesarean section; HR, Hazard ratio; NC, not calculated because of low number of events; VBAC, vaginal birth after previous caesarean

**Table S5. Complete case analysis of outcomes according to actual mode of birth – planned and actually had a VBAC and planned VBAC but had in-labour non-elective repeat caesarean section compared to ERCS**

|  | **Planned and actually had VBAC vs. ERCS** | | | |  | **Planned VBAC but had in-labour non-elective repeat caesarean section vs. ERCS** | | | |
| --- | --- | --- | --- | --- | --- | --- | --- | --- | --- |
|  | **Base model^1^ HR (95% CI)** | **Model A^2^ HR (95% CI)** | **Model B^3^ HR (95% CI)** | **Model C^4^ HR (95% CI)** |  | **Base model^1^ HR (95% CI)** | **Model A^2^ HR (95% CI)** | **Model B^3^ HR (95% CI)** | **Model C^4^ HR (95% CI)** |
| Dispensed any psychotropic medication | **0.80 (0.73 - 0.87)** | **0.79 (0.72 - 0.85)** | **0.80 (0.73 - 0.88)** | **0.83 (0.75 - 0.92)** |  | **0.82 (0.73 - 0.92)** | **0.85 (0.75 - 0.96)** | 1.00 (0.87 - 1.15) | 1.03 (0.89 - 1.20) |
|  | **P<0.001** | **P<0.001** | **P<0.001** | **P=0.001** |  | **P=0.001** | **P=0.007** | P=0.992 | P=0.666 |
| Dispensed an antidepressant | **0.76 (0.70 - 0.84)** | **0.75 (0.68 - 0.82)** | **0.76 (0.68 - 0.84)** | **0.79 (0.71 - 0.89)** |  | **0.83 (0.73 - 0.94)** | **0.85 (0.75 - 0.97)** | 1.00 (0.86 - 1.17) | 1.06 (0.90 - 1.24) |
|  | **P<0.001** | **P<0.001** | **P<0.001** | **P<0.001** |  | **P=0.004** | **P=0.017** | P=0.951 | P=0.503 |
| Dispensed at least two consecutive prescriptions for an antidepressant | **0.73 (0.66 - 0.81)** | **0.72 (0.65 - 0.80)** | **0.76 (0.67 - 0.86)** | **0.81 (0.71 - 0.92)** |  | **0.83 (0.71 - 0.96)** | 0.86 (0.74 - 1.00) | 1.02 (0.86 - 1.22) | 1.10 (0.92 - 1.32) |
|  | **P<0.001** | **P<0.001** | **P<0.001** | **P=0.002** |  | **P=0.013** | P=0.052 | P=0.791 | P=0.297 |
| Dispensed a hypnotic and/or anxiolytic | 0.89 (0.75 - 1.04) | 0.88 (0.75 - 1.04) | 0.93 (0.77 - 1.12) | 0.94 (0.77 - 1.15) |  | **0.78 (0.61 - 1.00)** | 0.83 (0.64 - 1.06) | 1.00 (0.75 - 1.34) | 1.03 (0.76 - 1.40) |
|  | P=0.137 | P=0.135 | P=0.431 | P=0.558 |  | **P=0.046** | P=0.130 | P=0.983 | P=0.841 |
| Dispensed an antipsychotic and/or related drug | 1.27 (0.71 - 2.28) | 1.22 (0.67 - 2.20) | NC | NC |  | **2.20 (1.12 - 4.32)** | **2.27 (1.14 - 4.52)** | NC | NC |
|  | P=0.417 | P=0.512 |  |  |  | **P=0.022** | **P=0.020** |  |  |

1 Base model adjusted for year of delivery only.

2 Model A adjusted for year of delivery and socio-demographic factors (maternal age, mother's country of birth, marital status and socio-economic status).

3 Model B adjusted for variables in Model A and additionally adjusted for maternal medical and pregnancy-related factors (number of previous caesarean sections, any prior vaginal birth, inter-pregnancy interval, any prior stillbirth or neonatal death, maternal smoking status at booking, maternal BMI at booking, hypertensive disorder, and diabetes).

4 Model C adjusted for variables in Model B and additionally adjusted for any breastfeeding at 6-8 weeks postpartum.

Bold text indicates statistically significant findings at the 5% level.

Abbreviations: CI, confidence interval; ERCS, elective repeat caesarean section; HR, Hazard ratio; NC, not calculated because of low number of events; VBAC, vaginal birth after previous caesarean

**Table S6. Outcomes following planned VBAC compared to ERCS at ≥ 39 weeks’ gestation**

| **Outcomes** | **ERCS** | **Planned VBAC** | **Base model^1^ HR (95% CI)** | **Model A^2^ HR (95% CI)** | **Model B^3^ HR (95% CI)** | **Model C^4^ HR (95% CI)** |
| --- | --- | --- | --- | --- | --- | --- |
|  | **Number of events/person-years (Rate per 100 person-years)** | **Number of events/person-years (Rate per 100 person-years)** |  |  |  |  |
| Dispensed any psychotropic medication | 1871/15052 (12.43) | 802/7762 (10.33) | **0.83 (0.77 - 0.91)** | **0.83 (0.77 - 0.90)** | **0.82 (0.74 - 0.89)** | **0.87 (0.79 - 0.95)** |
|  |  |  | **P<0.001** | **P<0.001** | **P<0.001** | **P=0.002** |
| Dispensed an antidepressant | 1629/15167 (10.74) | 678/7827 (8.66) | **0.81 (0.74 - 0.89)** | **0.81 (0.74 - 0.88)** | **0.79 (0.72 - 0.87)** | **0.84 (0.76 - 0.93)** |
|  |  |  | **P<0.001** | **P<0.001** | **P<0.001** | **P=0.001** |
| Dispensed at least two consecutive prescriptions for an antidepressant | 1250/15379 (8.13) | 491/7925 (6.20) | **0.77 (0.69 - 0.85)** | **0.77 (0.69 - 0.86)** | **0.76 (0.68 - 0.86)** | **0.82 (0.73 - 0.92)** |
|  |  |  | **P<0.001** | **P<0.001** | **P<0.001** | **P=0.001** |
| Dispensed a hypnotic and/or anxiolytic | 437/15848 (2.76) | 208/8075 (2.58) | 0.93 (0.79 - 1.10) | 0.95 (0.80 - 1.12) | 0.93 (0.78 - 1.11) | 0.99 (0.83 - 1.19) |
|  |  |  | P=0.385 | P=0.506 | P=0.419 | P=0.940 |
| Dispensed an antipsychotic and/or related drug | 20/16032 (0.12) | 20/8163 (0.25) | **1.97 (1.07 - 3.63)** | **1.94 (1.04 - 3.64)** | NC | NC |
|  |  |  | **P=0.030** | **P=0.038** |  |  |

1 Base model adjusted for year of delivery only.

2 Model A adjusted for year of delivery and socio-demographic factors (maternal age, mother's country of birth, marital status and socio-economic status).

3 Model B adjusted for variables in Model A and additionally adjusted for maternal medical and pregnancy-related factors (number of previous caesarean sections, any prior vaginal birth, inter-pregnancy interval, any prior stillbirth or neonatal death, maternal smoking status at booking, maternal BMI at booking, hypertensive disorder, and diabetes).

4 Model C, adjusted for variables in Model B and additionally adjusted for any breastfeeding at 6-8 weeks postpartum.

Bold text indicates statistically significant findings at the 5% level.

Abbreviations: CI, confidence interval; ERCS, elective repeat caesarean section; HR, Hazard ratio; NC, not calculated because of low number of events; VBAC, vaginal birth after previous caesarean

**Table S7. Outcomes following planned VBAC with and without labour induction compared to ERCS at ≥ 39 weeks’ gestation**

|  | **ERCS** | **Planned VBAC without labour induction** | | | | |  | **Planned VBAC with labour induction** | | | | |
| --- | --- | --- | --- | --- | --- | --- | --- | --- | --- | --- | --- | --- |
|  | **Number of events/ person-years (Rate per 100 person-years)** | **Number of events/ person-years (Rate per 100 person-years)** | **Base model^1^ HR (95% CI)** | **Model A^2^ HR (95% CI)** | **Model B^3^ HR (95% CI)** | **Model C^4^ HR (95% CI)** |  | **Number of events/ person-years (Rate per 100 person-years)** | **Base model^1^ HR (95% CI)** | **Model A^2^ HR (95% CI)** | **Model B^3^ HR (95% CI)** | **Model C^4^ HR (95% CI)** |
| Dispensed any psychotropic medication | 1871/15052 | 630/6221 | **0.82** | **0.82** | **0.81** | **0.87** |  | 169/1509 | 0.90 | 0.86 | **0.82** | 0.88 |
|  | (12.43) | (10.13) | **(0.75 - 0.89)** | **(0.75 - 0.90)** | **(0.74 - 0.90)** | **(0.78 - 0.96)** |  | (11.20) | (0.77 - 1.06) | (0.74 - 1.01) | **(0.70 - 0.97)** | (0.75 - 1.04) |
|  |  |  | **P<0.001** | **P<0.001** | **P<0.001** | **P=0.004** |  |  | P=0.199 | P=0.069 | **P=0.021** | P=0.126 |
| Dispensed an antidepressant | 1629/15167 | 529/6272 | **0.79** | **0.79** | **0.78** | **0.83** |  | 146/1522 | 0.89 | 0.85 | **0.82** | 0.87 |
|  | (10.74) | (8.43) | **(0.72 - 0.87)** | **(0.72 - 0.88)** | **(0.70 - 0.87)** | **(0.75 - 0.93)** |  | (9.59) | (0.75 - 1.06) | (0.72 - 1.01) | **(0.69 - 0.98)** | (0.73 - 1.04) |
|  |  |  | **P<0.001** | **P<0.001** | **P<0.001** | **P=0.001** |  |  | P=0.195 | P=0.061 | **P=0.026** | P=0.133 |
| Dispensed at least two consecutive prescriptions for an antidepressant | 1250/15379 | 388/6342 | **0.76** | **0.77** | **0.77** | **0.83** |  | 101/1551 | **0.80** | **0.77** | **0.75** | **0.80** |
|  | (8.13) | (6.12) | **(0.68 - 0.85)** | **(0.69 - 0.86)** | **(0.68 - 0.87)** | **(0.73 - 0.94)** |  | (6.51) | **(0.65 - 0.98)** | **(0.63 - 0.95)** | **(0.61 - 0.92)** | **(0.65 - 0.99)** |
|  |  |  | **P<0.001** | **P<0.001** | **P<0.001** | **P=0.003** |  |  | **P=0.034** | **P=0.013** | **P=0.007** | **P=0.043** |
| Dispensed a hypnotic and/or anxiolytic | 437/15848 | 165/6464 | 0.92 | 0.94 | 0.94 | 1.00 |  | 43/1577 | 0.99 | 0.97 | 0.91 | 0.98 |
|  | (2.76) | (2.55) | (0.77 - 1.10) | (0.79 - 1.13) | (0.77 - 1.14) | (0.83 - 1.22) |  | (2.73) | (0.72 - 1.35) | (0.71 - 1.33) | (0.66 - 1.27) | (0.71 - 1.36) |
|  |  |  | P=0.352 | P=0.527 | P=0.516 | P=0.978 |  |  | P=0.938 | P=0.867 | P=0.592 | P=0.910 |
| Dispensed an antipsychotic and/or related drug | 20/16032 | 16/6531 | **1.97** | **1.95** | NC | NC |  | 4/1598 | 2.01 | 1.94 | NC | NC |
|  | (0.12) | (0.24) | **(1.03 - 3.77)** | **(1.00 - 3.80)** |  |  |  | (0.25) | (0.69 - 5.87) | (0.66 - 5.74) |  |  |
|  |  |  | **P=0.040** | **P=0.049** |  |  |  |  | P=0.203 | P=0.229 |  |  |

1 Base model adjusted for year of delivery only.

2 Model A adjusted for year of delivery and socio-demographic factors (maternal age, mother's country of birth, marital status and socio-economic status).

3 Model B adjusted for variables in Model A and additionally adjusted for maternal medical and pregnancy-related factors (number of previous caesarean sections, any prior vaginal birth, inter-pregnancy interval, any prior stillbirth or neonatal death, maternal smoking status at booking, maternal BMI at booking, hypertensive disorder, and diabetes).

4 Model C, adjusted for variables in Model B and additionally adjusted for any breastfeeding at 6-8 weeks postpartum.

Bold text indicates statistically significant findings at the 5% level.

Abbreviations: CI, confidence interval; ERCS, elective repeat caesarean section; HR, Hazard ratio; NC, not calculated because of low number of events; VBAC, vaginal birth after previous caesarean

**Table S8. Outcomes according to actual mode of birth – planned and actually had a VBAC and planned VBAC but had in-labour non-elective repeat caesarean section compared to ERCS at ≥ 39 weeks’ gestation**

|  | **ERCS** | **Planned and actually had VBAC** | | | | |  | **Planned VBAC but had in-labour non-elective repeat caesarean section** | | | | |
| --- | --- | --- | --- | --- | --- | --- | --- | --- | --- | --- | --- | --- |
|  | **Number of events/ person-years (Rate per 100 person-years)** | **Number of events/ person-years (Rate per 100 person-years)** | **Base model^1^ HR (95% CI)** | **Model A^2^  HR (95% CI)** | **Model B^3^ HR (95% CI)** | **Model C^4^ HR (95% CI)** |  | **Number of events/ person-years (Rate per 100 person-years)** | **Base model^1^ HR (95% CI)** | **Model A^2^ HR (95% CI)** | **Model B^3^ HR (95% CI)** | **Model C^4^ HR (95% CI)** |
| Dispensed any psychotropic medication | 1871/15052 | 585/5560 | **0.85** | **0.84** | **0.81** | **0.86** |  | 217/2202 | **0.79** | **0.82** | **0.82** | 0.88 |
|  | (12.43) | (10.52) | **(0.77 - 0.93)** | **(0.76 - 0.92)** | **(0.73 - 0.90)** | **(0.78 - 0.96)** |  | (9.86) | **(0.69 - 0.91)** | **(0.71 - 0.95)** | **(0.71 - 0.94)** | (0.76 - 1.02) |
|  |  |  | **P<0.001** | **P<0.001** | **P<0.001** | **P=0.005** |  |  | **P=0.001** | **P=0.007** | **P=0.006** | P=0.079 |
| Dispensed an antidepressant | 1629/15167 | 487/5609 | **0.81** | **0.80** | **0.78** | **0.82** |  | 191/2218 | **0.80** | **0.83** | **0.82** | 0.89 |
|  | (10.74) | (8.68) | **(0.73 - 0.90)** | **(0.72 - 0.88)** | **(0.69 - 0.87)** | **(0.74 - 0.92)** |  | (8.61) | **(0.69 - 0.94)** | **(0.71 - 0.96)** | **(0.71 - 0.96)** | (0.76 - 1.03) |
|  |  |  | **P=0.001** | **P<0.001** | **P<0.001** | **P=0.001** |  |  | **P=0.005** | **P=0.015** | **P=0.013** | P=0.126 |
| Dispensed at least two consecutive prescriptions for an antidepressant | 1250/15379 | 349/5685 | **0.76** | **0.75** | **0.75** | **0.80** |  | 142/2240 | **0.78** | **0.82** | **0.81** | 0.88 |
|  | (8.13) | (6.14) | **(0.68 - 0.86)** | **(0.67 - 0.85)** | **(0.65 - 0.85)** | **(0.70 - 0.91)** |  | (6.34) | **(0.66 - 0.93)** | **(0.69 - 0.97)** | **(0.68 - 0.97)** | (0.74 - 1.05) |
|  |  |  | **P<0.001** | **P<0.001** | **P<0.001** | **P=0.001** |  |  | **P=0.006** | **P=0.025** | **P=0.021** | P=0.166 |
| Dispensed a hypnotic and/or anxiolytic | 437/15848 | 157/5787 | 0.98 | 0.97 | 0.96 | 1.02 |  | 51/2288 | 0.80 | 0.86 | 0.86 | 0.93 |
|  | (2.76) | (2.71) | (0.81 - 1.17) | (0.81 - 1.17) | (0.78 - 1.17) | (0.83 - 1.25) |  | (2.23) | (0.60 - 1.08) | (0.65 - 1.16) | (0.64 - 1.15) | (0.69 - 1.25) |
|  |  |  | P=0.797 | P=0.783 | P=0.672 | P=0.850 |  |  | P=0.142 | P=0.328 | P=0.315 | P=0.621 |
| Dispensed an antipsychotic and/or related drug | 20/16032 | 12/5853 | 1.65 | 1.60 | NC | NC |  | 8/2310 | **2.78** | **2.87** | NC | NC |
|  | (0.12) | (0.21) | (0.82 - 3.32) | (0.78 - 3.27) |  |  |  | (0.35) | **(1.22 - 6.32)** | **(1.24 - 6.66)** |  |  |
|  |  |  | P=0.163 | P=0.199 |  |  |  |  | **P=0.015** | **P=0.014** |  |  |

1 Base model adjusted for year of delivery only.

2 Model A adjusted for year of delivery and socio-demographic factors (maternal age, mother's country of birth, marital status and socio-economic status).

3 Model B adjusted for variables in Model A and additionally adjusted for maternal medical and pregnancy-related factors (number of previous caesarean sections, any prior vaginal birth, inter-pregnancy interval, any prior stillbirth or neonatal death, maternal smoking status at booking, maternal BMI at booking, hypertensive disorder, and diabetes).

4 Model C, adjusted for variables in Model B and additionally adjusted for any breastfeeding at 6-8 weeks postpartum.

Bold text indicates statistically significant findings at the 5% level.

Abbreviations: CI, confidence interval; ERCS, elective repeat caesarean section; HR, Hazard ratio; NC, not calculated because of low number of events; VBAC, vaginal birth after previous caesarean

**Table S9. Outcomes following planned VBAC compared to ERCS in women without a history of being dispensed psychotropic drugs in the two years before delivery**

| **Outcomes** | **ERCS** | **Planned VBAC** | **Base model^1^ HR (95% CI)** | **Model A^2^ HR (95% CI)** | **Model B^3^ HR (95% CI)** | **Model C^4^ HR (95% CI)** |
| --- | --- | --- | --- | --- | --- | --- |
|  | **Number of events/person-years (Rate per 100 person-years)** | **Number of events/person-years (Rate per 100 person-years)** |  |  |  |  |
| Dispensed any psychotropic medication | 1600/15096 (10.60) | 645/7394 (8.72) | **0.83 (0.75 - 0.91)** | **0.82 (0.75 - 0.90)** | **0.82 (0.74 - 0.91)** | **0.87 (0.79 - 0.96)** |
|  |  |  | **P<0.001** | **P<0.001** | **P=0.001** | **P=0.007** |
| Dispensed an antidepressant | 1381/15196 (9.09) | 546/7442 (7.34) | **0.81 (0.74 - 0.90)** | **0.81 (0.73 - 0.89)** | **0.80 (0.72 - 0.89)** | **0.85 (0.76 - 0.95)** |
|  |  |  | **P<0.001** | **P<0.001** | **P<0.001** | **P=0.004** |
| Dispensed at least two consecutive prescriptions for an antidepressant | 1060/15370 (6.90) | 410/7517 (5.45) | **0.80 (0.71 - 0.89)** | **0.80 (0.71 - 0.89)** | **0.81 (0.72 - 0.91)** | **0.86 (0.76 - 0.98)** |
|  |  |  | **P<0.001** | **P<0.001** | **P=0.001** | **P=0.019** |
| Dispensed a hypnotic and/or anxiolytic | 381/15751 (2.42) | 160/7651 (2.09) | 0.87 (0.72 - 1.04) | 0.88 (0.73 - 1.06) | 0.86 (0.71 - 1.05) | 0.91 (0.75 - 1.11) |
|  |  |  | P=0.125 | P=0.173 | P=0.131 | P=0.354 |
| Dispensed an antipsychotic and/or related drug | 14/15910 (0.09) | 15/7714 (0.19) | **2.20 (1.07 - 4.53)** | **2.11 (1.01 - 4.42)** | NC | NC |
|  |  |  | **P=0.032** | **P=0.047** |  |  |

1 Base model adjusted for year of delivery only.

2 Model A adjusted for year of delivery and socio-demographic factors (maternal age, mother's country of birth, marital status and socio-economic status).

3 Model B adjusted for variables in Model A and additionally adjusted for maternal medical and pregnancy-related factors (number of previous caesarean sections, any prior vaginal birth, inter-pregnancy interval, any prior stillbirth or neonatal death, maternal smoking status at booking, maternal BMI at booking, hypertensive disorder, and diabetes).

4 Model C, adjusted for variables in Model B and additionally adjusted for any breastfeeding at 6-8 weeks postpartum.

Bold text indicates statistically significant findings at the 5% level.

Abbreviations: CI, confidence interval; ERCS, elective repeat caesarean section; HR, Hazard ratio; NC, not calculated because of low number of events; VBAC, vaginal birth after previous caesarean

**Table S10. Outcomes following planned VBAC with and without labour induction compared to ERCS in women without a history of being dispensed psychotropic drugs in the two years before delivery**

|  | **ERCS** | **Planned VBAC without labour induction** | | | | |  | **Planned VBAC with labour induction** | | | | |
| --- | --- | --- | --- | --- | --- | --- | --- | --- | --- | --- | --- | --- |
|  | **Number of events/ person-years (Rate per 100 person-years)** | **Number of events/ person-years (Rate per 100 person-years)** | **Base model^1^ HR (95% CI)** | **Model A^2^ HR (95% CI)** | **Model B^3^  HR (95% CI)** | **Model C^4^ HR (95% CI)** |  | **Number of events/ person-years (Rate per 100 person-years)** | **Base model^1^ HR (95% CI)** | **Model A^2^ HR (95% CI)** | **Model B^3^ HR (95% CI)** | **Model C^4^ HR (95% CI)** |
| Dispensed any psychotropic medication | 1600/15096 | 499/5909 | **0.80** | **0.80** | **0.81** | **0.86** |  | 143/1450 | 0.93 | 0.90 | 0.87 | 0.92 |
|  | (10.60) | (8.45) | **(0.72 - 0.89)** | **(0.73 - 0.89)** | **(0.73 - 0.90)** | **(0.77 - 0.96)** |  | (9.86) | (0.78 - 1.11) | (0.75 - 1.07) | (0.72 - 1.03) | (0.77 - 1.10) |
|  |  |  | **P<0.001** | **P<0.001** | **P=0.001** | **P=0.006** |  |  | P=0.409 | P=0.213 | P=0.112 | P=0.357 |
| Dispensed an antidepressant | 1381/15196 | 417/5948 | **0.78** | **0.78** | **0.78** | **0.83** |  | 126/1459 | 0.95 | 0.91 | 0.88 | 0.94 |
|  | (9.09) | (7.01) | **(0.70 - 0.87)** | **(0.70 - 0.87)** | **(0.69 - 0.88)** | **(0.74 - 0.93)** |  | (8.64) | (0.79 - 1.14) | (0.76 - 1.09) | (0.73 - 1.07) | (0.78 - 1.14) |
|  |  |  | **P<0.001** | **P<0.001** | **P<0.001** | **P=0.002** |  |  | P=0.581 | P=0.312 | P=0.201 | P=0.523 |
| Dispensed at least two consecutive prescriptions for an antidepressant | 1060/15370 | 311/6002 | **0.76** | **0.77** | **0.78** | **0.83** |  | 97/1479 | 0.95 | 0.92 | 0.91 | 0.97 |
|  | (6.90) | (5.18) | **(0.67 - 0.86)** | **(0.67 - 0.87)** | **(0.68 - 0.90)** | **(0.73 - 0.96)** |  | (6.56) | (0.77 - 1.17) | (0.74 - 1.13) | (0.73 - 1.13) | (0.78 - 1.20) |
|  |  |  | **P<0.001** | **P<0.001** | **P<0.001** | **P=0.009** |  |  | P=0.630 | P=0.432 | P=0.392 | P=0.774 |
| Dispensed a hypnotic and/or anxiolytic | 381/15751 | 127/6105 | 0.86 | 0.88 | 0.87 | 0.93 |  | 33/1510 | 0.90 | 0.90 | 0.83 | 0.88 |
|  | (2.42) | (2.08) | (0.70 - 1.05) | (0.72 - 1.08) | (0.71 - 1.08) | (0.75 - 1.15) |  | (2.19) | (0.63 - 1.29) | (0.63 - 1.28) | (0.57 - 1.19) | (0.61 - 1.26) |
|  |  |  | P=0.145 | P=0.217 | P=0.216 | P=0.483 |  |  | P=0.576 | P=0.548 | P=0.302 | P=0.481 |
| Dispensed an antipsychotic and/or related drug | 14/15910 | 12/6153 | **2.21** | 2.14 | NC | NC |  | 3/1524 | 2.24 | 2.05 | NC | NC |
|  | (0.09) | (0.20) | **(1.03 - 4.73)** | (0.99 - 4.64) |  |  |  | (0.20) | (0.64 - 7.80) | (0.57 - 7.44) |  |  |
|  |  |  | **P=0.042** | P=0.053 |  |  |  |  | P=0.206 | P=0.273 |  |  |

1 Base model adjusted for year of delivery only.

2 Model A adjusted for year of delivery and socio-demographic factors (maternal age, mother's country of birth, marital status and socio-economic status).

3 Model B adjusted for variables in Model A and additionally adjusted for maternal medical and pregnancy-related factors (number of previous caesarean sections, any prior vaginal birth, inter-pregnancy interval, any prior stillbirth or neonatal death, maternal smoking status at booking, maternal BMI at booking, hypertensive disorder, and diabetes).

4 Model C, adjusted for variables in Model B and additionally adjusted for any breastfeeding at 6-8 weeks postpartum.

Bold text indicates statistically significant findings at the 5% level.

Abbreviations: CI, confidence interval; ERCS, elective repeat caesarean section; HR, Hazard ratio; NC, not calculated because of low number of events; VBAC, vaginal birth after previous caesarean

**Table S11. Outcomes according to actual mode of birth – planned and actually had a VBAC and planned VBAC but had in-labour non-elective repeat caesarean section compared to ERCS in women without history of being dispensed psychotropic drugs in the two years before delivery**

|  | **ERCS** | **Planned and actually had VBAC** | | | | |  | **Planned VBAC but had in-labour non-elective repeat caesarean section** | | | | |
| --- | --- | --- | --- | --- | --- | --- | --- | --- | --- | --- | --- | --- |
|  | **Number of events/ person-years (Rate per 100 person-years)** | **Number of events/ person-years (Rate per 100 person-years)** | **Base model^1^ HR (95% CI)** | **Model A^2^ HR (95% CI)** | **Model B^3^ HR (95% CI)** | **Model C^4^ HR (95% CI)** |  | **Number of events/ person-years (Rate per 100 person-years)** | **Base model^1^ HR (95% CI)** | **Model A^2^ HR (95% CI)** | **Model B^3^ HR (95% CI)** | **Model C^4^ HR (95% CI)** |
| Dispensed any psychotropic medication | 1600/15096 | 454/5256 | **0.82** | **0.81** | **0.80** | **0.84** |  | 191/2138 | **0.84** | 0.87 | 0.87 | 0.94 |
|  | (10.60) | (8.64) | **(0.74 - 0.91)** | **(0.73 - 0.89)** | **(0.71 - 0.89)** | **(0.75 - 0.94)** |  | (8.93) | **(0.73 - 0.98)** | (0.75 - 1.01) | (0.75 - 1.02) | (0.80 - 1.09) |
|  |  |  | **P<0.001** | **P<0.001** | **P=0.001** | **P=0.003** |  |  | **P=0.028** | P=0.068 | P=0.083 | P=0.405 |
| Dispensed an antidepressant | 1381/15196 | 377/5291 | **0.79** | **0.77** | **0.76** | **0.81** |  | 169/2150 | 0.87 | 0.89 | 0.89 | 0.96 |
|  | (9.09) | (7.12) | **(0.70 - 0.88)** | **(0.69 - 0.87)** | **(0.67 - 0.86)** | **(0.71 - 0.91)** |  | (7.86) | (0.74 - 1.02) | (0.76 - 1.05) | (0.76 - 1.05) | (0.82 - 1.13) |
|  |  |  | **P<0.001** | **P<0.001** | **P<0.001** | **P<0.001** |  |  | P=0.083 | P=0.156 | P=0.179 | P=0.648 |
| Dispensed at least two consecutive prescriptions for an antidepressant | 1060/1537 | 277/5349 | **0.76** | **0.75** | **0.75** | **0.80** |  | 133/2168 | 0.89 | 0.93 | 0.93 | 1.01 |
|  | (6.90) | (5.18) | **(0.66 - 0.86)** | **(0.65 - 0.85)** | **(0.65 - 0.87)** | **(0.69 - 0.92)** |  | (6.13) | (0.75 - 1.07) | (0.77 - 1.11) | (0.78 - 1.12) | (0.84 - 1.21) |
|  |  |  | **P<0.001** | **P<0.001** | **P<0.001** | **P=0.002** |  |  | P=0.220 | P=0.417 | P=0.470 | P=0.943 |
| Dispensed a hypnotic and/or anxiolytic | 381/15751 | 121/5433 | 0.92 | 0.92 | 0.90 | 0.95 |  | 39/2219 | 0.73 | 0.77 | 0.76 | 0.81 |
|  | (2.42) | (2.23) | (0.75 - 1.13) | (0.75 - 1.13) | (0.72 - 1.13) | (0.76 - 1.19) |  | (1.76) | (0.52 - 1.01) | (0.55 - 1.07) | (0.54 - 1.06) | (0.58 - 1.13) |
|  |  |  | P=0.436 | P=0.446 | P=0.368 | P=0.679 |  |  | P=0.058 | P=0.115 | P=0.103 | P=0.215 |
| Dispensed an antipsychotic and/or related drug | 14/15910 | 9/5479 | 1.86 | 1.75 | NC | NC |  | 6/2235 | **3.04** | **3.06** | NC | NC |
|  | (0.09) | (0.16) | (0.81 - 4.25) | (0.75 - 4.07) |  |  |  | (0.27) | **(1.17 - 7.91)** | **(1.16 - 8.08)** |  |  |
|  |  |  | P=0.142 | P=0.192 |  |  |  |  | **P=0.022** | **P=0.024** |  |  |

1 Base model adjusted for year of delivery only.

2 Model A adjusted for year of delivery and socio-demographic factors (maternal age, mother's country of birth, marital status and socio-economic status).

3 Model B adjusted for variables in Model A and additionally adjusted for maternal medical and pregnancy-related factors (number of previous caesarean sections, any prior vaginal birth, inter-pregnancy interval, any prior stillbirth or neonatal death, maternal smoking status at booking, maternal BMI at booking, hypertensive disorder, and diabetes).

4 Model C, adjusted for variables in Model B and additionally adjusted for any breastfeeding at 6-8 weeks postpartum.

Bold text indicates statistically significant findings at the 5% level.

Abbreviations: CI, confidence interval; ERCS, elective repeat caesarean section; HR, Hazard ratio; NC, not calculated because of low number of events; VBAC, vaginal birth after previous caesarean

**Table S12. E-values for the observed associations between planned mode of birth after previous caesarean section (planned VBAC vs. ERCS) and outcomes***

| **Outcome** | **E-value for HR point estimate** | **E-value for upper limit of HR 95% CI** |
| --- | --- | --- |
| Dispensed any psychotropic medication | 1.63 | 1.39 |
| Dispensed an antidepressant | 1.70 | 1.46 |
| Dispensed at least two consecutive prescriptions for an antidepressant | 1.70 | 1.43 |

*The observed associations are the fully adjusted HRs shown in Table 2. See VanderWeele and Ding (VanderWeele & Ding, 2017) for formula used to calculate E-values.

Abbreviations: CI, confidence interval; ERCS, elective repeat caesarean section; HR, Hazard ratio; VBAC, vaginal birth after previous caesarean

**References**

**Information Services Division Scotland**. Data dictionary SMR02 Maternity Inpatient and Day Case. Retrieved from <https://www.ndc.scot.nhs.uk/Data-Dictionary/SMR-Datasets/SMR02-Maternity-Inpatient-and-Day-Case/>. Accessed 10 January 2019

**Information Services Division Scotland**. (2010). *Data Quality Assurance Assessment of Maternity Data (SMR02) 2008-2009* Retrieved from: <https://www.isdscotland.org/data_quality_assurance/DQA-Assessment-of-Maternity-Data-SMR02-2008-to-2009.pdf>. Accessed 10 Jan 2019

**Information Services Division Scotland**. (2012). *Assessment of SMR01 Data 2010 – 2011*. Retrieved from: <http://www.isdscotland.org/Health-Topics/Hospital-Care/Publications/2012-05-08/Assessment-of-SMR01Data-2010-2011-ScotlandReport.pdf>. Accessed 10 January 2019

**Information Services Division Scotland**. (2016). *Prescribing & Medicines: Medicines used in Mental Health - Years 2005/06 to 2015/16*. Retrieved from: <https://isdscotland.scot.nhs.uk/Health-Topics/Prescribing-and-Medicines/Publications/2016-10-04/2016-10-04-PrescribingMentalHealth-Report.pdf> Accessed 16 May 2018

**Information Services Division Scotland**. (2017a). *Births in Scottish Hospitals Technical Report Publication date - 28 November 2017*. Retrieved from: <http://www.isdscotland.org/Health-Topics/Maternity-and-Births/Publications/2017-11-28/2017-11-28-Births-Technical.pdf> Accessed 12 March 2018

**Information Services Division Scotland**. (2017b). *Infant Feeding Statistics Scotland Publication date - 31 October 2017*. Retrieved from: <https://www.isdscotland.org/Health-Topics/Child-Health/Publications/2017-10-31/2017-10-31-Infant-Feeding-Report.pdf> Accessed 12 March 2018

**Information Services Division Scotland**. (2018). Medicines Used in Mental Health - BNF Legacy. Retrieved from <https://www.isdscotland.org/Health-Topics/Prescribing-and-medicines/Community-Dispensing/Mental-Health/>

**Information Services Division Scotland**. (2019). *Assessment of SMR02 (Maternity Inpatient and Day Case) Data Scotland 2017-2018*. Retrieved from: <https://www.isdscotland.org/Products-and-Services/Data-Quality/docs/20191023-Assessment-of-SMR02-Data-Scotland-2017-2018.pdf>. Accessed 24 September 2020

**Knight, M., Kurinczuk, J. J., Spark, P., Brocklehurst, P., & UKOSS.** (2010). Extreme obesity in pregnancy in the United Kingdom. *Obstetrics & Gynecology, 115*(5), 989-997. doi:10.1097/AOG.0b013e3181da8f09

**National Records of Scotland**. Quality of Data Obtained from the Registration of Births, Stillbirths, Marriages, Civil Partnerships and Deaths. Retrieved from <https://www.nrscotland.gov.uk/files//statistics/vital-events/quality-data-obtained-from-registration-of-ve.pdf>. Accessed 25 September 2020

**VanderWeele, T. J., & Ding, P.** (2017). Sensitivity Analysis in Observational Research: Introducing the E-Value. *Annals of Internal Medicine, 167*(4), 268-274. doi:10.7326/M16-2607
